# Transmission of drug-resistant bacteria in a hospital-community model stratified by patient risk

**DOI:** 10.1038/s41598-023-45248-3

**Published:** 2023-10-30

**Authors:** Paweł Brachaczek, Agata Lonc, Mirjam E. Kretzschmar, Rafael Mikolajczyk, Johannes Horn, Andre Karch, Konrad Sakowski, Monika J. Piotrowska

**Affiliations:** 1 Faculty of Mathematics, Informatics and Mechanics, University of Warsaw, Banacha 2, 02-097 Warsaw, Poland; 2 Julius Center for Health Sciences and Primary Care, University Medical Center Utrecht, Utrecht University, Utrecht, The Netherlands; 3 Institute for Medical Epidemiology, Biometry, and Informatics (IMEBI), Interdisciplinary Center for Health Sciences, Medical Faculty of the Martin Luther University Halle Wittenberg, Halle (Saale), Germany; 4 Institute of Epidemiology and Social Medicine, University of Münster, Münster, Germany; 5 Institute of Applied Mathematics and Mechanics, University of Warsaw, Banacha 2, 02-097 Warsaw, Poland

**Keywords:** Applied mathematics, Epidemiology

## Abstract

A susceptible-infectious-susceptible (SIS) model for simulating healthcare-acquired infection spread within a hospital and associated community is proposed. The model accounts for the stratification of in-patients into two susceptibility-based risk groups. The model is formulated as a system of first-order ordinary differential equations (ODEs) with appropriate initial conditions. The mathematical analysis of this system is demonstrated. It is shown that the system has unique global solutions, which are bounded and non-negative. The basic reproduction number ($\mathscr {R}_0$) for the considered model is derived. The existence and the stability of the stationary solutions are analysed. The disease-free stationary solution is always present and is globally asymptotically stable for $\mathscr {R}_0<1$, while for $\mathscr {R}_0>1$ it is unstable. The presence of an endemic stationary solution depends on the model parameters and when it exists, it is globally asymptotically stable. The endemic state encompasses both risk groups. The endemic state within only one group only is not possible. In addition, for $\mathscr {R}_0=1$ a forward bifurcation takes place. Numerical simulations, based on the anonymised insurance data, are also presented to illustrate theoretical results.

## Introduction

An important step paving the road for modern medicine was the discovery of antibiotics. Unfortunately, due to evolutionary processes, the susceptibility of microorganisms to antibiotics diminishes with time. Widespread use of antibiotics intensifies this process and leads to the emergence of antibiotic-resistant bacteria^1–3^ and to the need for the investigation of the impact of antibiotic use on mortality^4^. Multiresistant pathogens are often spread within hospital networks by transfers or readmissions of colonised patients from the community^5–7^. In recent years, multiple modelling studies were conducted to assess the extent of the problem and introduce potential interventions^8–10^. Modelling studies were most often based on admission and discharge data, which also might have contained information on diagnoses of the patients^11–19^. Nevertheless, these previous research generally did not consider differences between individual patients but rather focused only on patient streams between institutions. In consequence, all patients were considered the same.

On the other hand, the considered model belongs to a family of so-called compartmental patch SIS models^20–22^ or multi-group SIS models^23–25^. In contrast to^22–25^, where incidence is modelled by bilinear term, we assume non-linear dependence. Moreover, in modelling our problem, we cannot assume that the connectivity matrix is symmetric as in^20^, but rather we should consider an asymmetric matrix following^21^. Since the interacting individuals are stratified into low- and high-risk groups and we also model the screening process, the model structure of previously considered models is violated.

In reality, patients differ in their risk of becoming colonised during a hospital stay. The risk of colonisation depends on a patient’s diagnosis, since it determines their comorbidities and antibiotic use, as well as which hospital ward the patient is admitted to, how frequent their contacts with healthcare workers are, and how long they stay in a hospital. In addition, the diagnoses and comorbidities influence how often a patient is readmitted to a hospital. Patients’ diagnoses are stored in hospital records as ICD-10 codes and can be used for risk stratification. This was first done in^26^, where the authors introduced a risk-stratified transmission model within hospitals. Heterogeneity in the risk of colonisation likely influences the transmission dynamics of resistant bacteria in hospitals and, through patient transfers, in the entire healthcare system. In order to design effective interventions, it is therefore essential to understand the impact of risk heterogeneity on the dynamics of resistant bacteria transmission.

Here, we extend the previously published model for the hospital-community pairs^17^ by adding risk stratification of patients. We aim to investigate the impact of risk heterogeneity on the transmission dynamics within single disjoint hospital-community pairs. We use data from a German health insurance company to determine the parameters of the model. We first present analytical results for an 8-dimensional system of ODEs describing a hospital-community pair, followed by simulation results discussing the effectiveness of the considered interventions. In particular, we discuss the effectiveness of transmission rates reduction in specific risk groups and the effectiveness of screening.

## Single hospital-community pair model taking into account patient risk groups

The spread of bacteria within a single hospital-community pair can be modelled by a modified version of a susceptible-infectious-susceptible (SIS) model, considered e.g. in Piotrowska et al.^17^. To distinguish two patient risk groups, we introduce additional variables to the model, indexed by $i\in \{1,2\}$. Here $i=1$ denotes the low-risk group and $i=2$ the high-risk group.

For each patient risk group *i*, we define the following variables as fractions of the total population:

susceptible individuals in the hospital $S_i,$ colonised individuals in the hospital $I_i$, susceptible individuals in the community $V_i,$ and colonised individuals in the community $W_i$, and thus $\sum _{i = 1}^2 (S_i+I_i+V_i+W_i)=1$. We assume that pathogen transmission can occur only in the hospital (c.f.^17,18^), while the clearance of colonisation takes place in both the hospital and the community. Susceptible individuals (in the hospital) of group *i* are exposed equally to colonised individuals of both risk groups. Moreover, following^26^, we assign patients to risk groups according to their medical history, so that they do not migrate between risk groups, for details see section “Patient stratification and parameter estimation”.

Thus, we assume that model parameters depend on risk group *i*, and as a consequence parameter $\beta _i > 0$ denotes the susceptibility-based transmission rate for *i*-th risk group, while the corresponding clearance rate is denoted by $\gamma _i > 0.$

Patients are discharged from the hospital at rate $\alpha _i$ and readmitted at rate $\varepsilon _i,$ where $\alpha _i, \varepsilon _i \in (0,1].$ Furthermore, we assume that patients are screened at admission, and if they are found positive, they are decolonised with probability $0 \le \sigma < 1$ and then enter the hospital as susceptible.

The process considered above can be described by the following system of ODEs:

1a $$\begin{aligned} \frac{\mathop {}\!\textrm{d}S_i}{\mathop {}\!\textrm{d}t}&= -\beta _i \frac{I}{I+S} S_i - \alpha _i S_i + \gamma _i I_i + \varepsilon _i V_i + \sigma \varepsilon _i W_i , \end{aligned}$$

1b $$\begin{aligned} \frac{\mathop {}\!\textrm{d}I_i}{\mathop {}\!\textrm{d}t}&= \beta _i \frac{I}{I+S} S_i - \alpha _i I_i - \gamma _i I_i + (1 - \sigma ) \varepsilon _i W_i , \end{aligned}$$

1c $$\begin{aligned} \frac{\mathop {}\!\textrm{d}V_i}{\mathop {}\!\textrm{d}t}&= \alpha _i S_i - \varepsilon _i V_i + \gamma _i W_i , \end{aligned}$$

1d $$\begin{aligned} \frac{\mathop {}\!\textrm{d}W_i}{\mathop {}\!\textrm{d}t}&= \alpha _i I_i - \varepsilon _i W_i - \gamma _i W_i \end{aligned}$$

for $i=1,2,$ where $S = S_1 + S_2$, $I=I_1+I_2$. Terms $\beta _i \frac{I}{I+S} S_i$ and $\gamma _i I_i$, $\gamma _i W_i$ represent the processes of colonisation of susceptible patients and decolonisation (recovery) of colonised patients, respectively. Terms $\alpha _i S_i, \alpha _i I_i$ describe discharge of susceptible and colonised patients, and terms $\varepsilon _i V_i, \varepsilon _i W_i$ describe admission of susceptible and colonised individuals from the community, respectively. Term $\sigma \varepsilon _i W_i$ describes screening and further decolonisation of colonised individuals at the admission, while the term $(1-\sigma ) \varepsilon _i W_i$ describes admission of colonised patients who are not successfully decolonised yet.

To simplify the notation, we write $G_i = S_i + I_i + V_i + W_i$

where $G_1$ and $G_2$ are constants. As all the variables denote fractions of the population, we have $S_1 + S_2 + I_1 + I_2 + V_1 + V_2 + W_1 + W_2 = 1$ and $G_1 + G_2 = 1$. In addition, the fractions of individuals from the *i*-th group in the hospital and the community are denoted by

$$\begin{aligned} H_i = S_i + I_i \qquad \text {and} \qquad C_i = V_i + W_i, \end{aligned}$$

respectively. To close system (1), we assume that initial conditions satisfy:

2 $$\begin{aligned} S_i(0), I_i(0), V_i(0), W_i(0) \ge 0&\qquad \text {for} \qquad i=1,2, \nonumber \\ S_i(0) + I_i(0) + V_i(0) + W_i(0) = G_i&\qquad \text {for} \qquad i=1,2, \nonumber \\ H_i(0), C_i(0) > 0&\qquad \text {for} \qquad i=1,2. \end{aligned}$$

### Mathematical properties of the single hospital-community pair model with patient risk groups

First, we focus on the basic properties of the solutions of the considered system.

#### Statement 1

System (1)–(2) has global and unique solutions which are non-negative and bounded from above by 1.

#### Proof

Since the right-hand side of system (1) is continuous with respect to *t* and locally Lipschitz continuous with respect to $S_i, I_i, V_i, W_i$, system (1)–(2) has local and unique solutions as a direct consequence of Picard-Lindelöf theorem.

Let us observe that for $t\ge 0$

3a $$\begin{aligned} H_i(t)&=\frac{\varepsilon _i}{\alpha _i + \varepsilon _i} G_i +e^{-(\alpha _i+\varepsilon _i)t}\left( H_i(0)-\frac{\varepsilon _i}{\alpha _i + \varepsilon _i} G_i \right) >0, \end{aligned}$$

3b $$\begin{aligned} C_i(t)&=\frac{\alpha _i}{\alpha _i + \varepsilon _i} G_i +e^{-(\alpha _i+\varepsilon _i)t}\left( C_i(0)-\frac{\alpha _i}{\alpha _i + \varepsilon _i} G_i \right) >0, \end{aligned}$$

so since $S(t)+I(t)=H_1(t)+H_2(t)$, the right-hand side of (1) is a smooth function on the interval of existence of solutions.

In order to prove the non-negativity of the solutions, we recall the Taylor formula. Let $$g\in C^n$$ be a function defined on an interval $$[0,t_1)$$ such that $$g(0)\ge 0$$. By $$0\le t_0<t_1$$ we denote the first time point at which *g* is equal to 0. Then

4 $$\begin{aligned} g(t)=\sum _{i=0}^{m}\frac{(t-t_0)^i}{i!}\frac{\mathop {}\!\textrm{d}^i g(t_0)}{\mathop {}\!\textrm{d}t^{i}} + R_m(t,t_0), \end{aligned}$$

where $m\le n-1$ and $\lim _{t\rightarrow t_0} \frac{R_m(t,t_0)}{(t-t_0)^m}=0$. Let *k* be the index of the first non-zero derivative of *g* at point $t_0$. If $\frac{\mathop {}\!\textrm{d}^k g(t_0)}{\mathop {}\!\textrm{d}t^k}$ is positive, then there exists $\delta >0$ such that for all $t\in (t_0,t_0+\delta )$ we have

$$\begin{aligned} g(t) = \frac{(t-t_0)^k}{k!}\frac{\mathop {}\!\textrm{d}^k g(t_0)}{\mathop {}\!\textrm{d}t^{k}} + R_k(t,t_0)>0. \end{aligned}$$

Then we can repeat this proof for any subsequent roots of *g*.

Consider system (1)–(2). Let $t_0\ge 0$ denote the first time point at which any of the variables $S_i(t)$, $I_i(t)$, $V_i(t)$, $W_i(t)$, $i=1,2$ (possibly more than one) is equal to 0 while the remaining variables are positive.

If $S_i(t_0)=0$, then from (3a) we have $I_i(t_0)=H_i(t_0)>0$ and thus

$$\begin{aligned} \frac{\mathop {}\!\textrm{d}S_i(t_0)}{\mathop {}\!\textrm{d}t} \ge \gamma _i I_i(t_0)>0. \end{aligned}$$

Similarly, if $V_i(t_0)=0$, then (3b) implies that $W_i(t_0)=C_i(t_0)>0$ and

$$\begin{aligned} \frac{\mathop {}\!\textrm{d}V_i(t_0)}{\mathop {}\!\textrm{d}t} \ge \gamma _i W_i(t_0)>0. \end{aligned}$$

If $W_i(t_0)=0$ then the sign of $\frac{\mathop {}\!\textrm{d}W_i(t_0)}{\mathop {}\!\textrm{d}t}$ is the same as the sign of $I_i(t_0)$. For $I_i(t_0)=0$, conditions $W_i(t_0)>0$ or $I_{3-i}(t_0)>0$ yield $\frac{\mathop {}\!\textrm{d}I_i(t_0)}{\mathop {}\!\textrm{d}t}>0$. If both $W_i(t_0)=0$ and $I_i(t_0)=0$, then

$$\begin{aligned} \frac{\mathop {}\!\textrm{d}^2W_i(t_0)}{\mathop {}\!\textrm{d}t^2}=\alpha _i\frac{\mathop {}\!\textrm{d}I_i(t_0)}{\mathop {}\!\textrm{d}t} = \alpha _i\beta _i\frac{I_{3-i}(t_0)}{I_{3-i}(t_0)+S(t_0)}S_i(t_0), \end{aligned}$$

so $\frac{\mathop {}\!\textrm{d}^2W_i(t_0)}{\mathop {}\!\textrm{d}t^2}$ and $\frac{\mathop {}\!\textrm{d}I_i(t_0)}{\mathop {}\!\textrm{d}t}$ both have the same sign as $I_{3-i}(t_0)$. If additionally $I_{3-i}(t_0)=0$ and $W_{3-i}(t_0)>0$, then from previous observation we have $\frac{\mathop {}\!\textrm{d}I_{3-i}(t_0)}{\mathop {}\!\textrm{d}t}>0$. Furthermore,

$$\begin{aligned} \frac{\mathop {}\!\textrm{d}^3 W_i(0)}{\mathop {}\!\textrm{d}t^{3}} = \alpha _i\frac{\mathop {}\!\textrm{d}^2 I_{i}(t_0)}{\mathop {}\!\textrm{d}t^2} = \alpha _i\beta _i\frac{S_i(t_0)}{S(t_0)}\frac{\mathop {}\!\textrm{d}I_{3-i}(t_0)}{\mathop {}\!\textrm{d}t}>0. \end{aligned}$$

If all $I_1(t_0)$, $I_2(t_0)$, $W_1(t_0)$, $W_2(t_0)$ are equal to 0, then $I_1(0)$, $I_2(0)$, $W_1(0)$, $W_2(0)$ are also all equal to 0. Otherwise, the solution of (1)–(2) would intersect with a solution $(\widetilde{S}_1,\widetilde{I}_1,\widetilde{V}_1,\widetilde{W}_1,\widetilde{S}_2,\widetilde{I}_2,\widetilde{V}_2,\widetilde{W}_2)$ describing a population without any colonised patients, i.e.

$$\begin{aligned} \begin{aligned} \frac{\mathop {}\!\textrm{d}\widetilde{S}_i}{\mathop {}\!\textrm{d}t}&= - \alpha _i \widetilde{S}_i + \varepsilon _i \widetilde{V}_i,\\ \frac{\mathop {}\!\textrm{d}\widetilde{V}_i}{\mathop {}\!\textrm{d}t}&= \alpha _i \widetilde{S}_i - \varepsilon _i \widetilde{V}_i,\\ \widetilde{I}_i(t)&=0, \quad \widetilde{W}_i(t)=0, \end{aligned} \end{aligned}$$

for $i=1,2$ with initial conditions $\widetilde{S}_i(0)=S_i(t_0)$, $\widetilde{V}_i(0)=V_i(t_0)$, $i=1,2$. Since we proved that the solutions are unique, such a situation cannot take place. If $I_1(0)$, $I_2(0)$, $W_1(0)$, $W_2(0)$ are all equal to 0, then the solutions of system (1)–(2) are non-negative from (3), since $S_i(t)=H_i(t)$ and $V_i(t)=C_i(t)$.

Thus, $S_i(t), I_i(t), V_i(t), W_i(t) \in [0,1]$ for every *t* from the interval of existence. From this observation and the fact that $S(t)+I(t)>0$, it is trivial to check that the right-hand side of system (1) is bounded. Hence, solutions of system (1)–(2) are bounded functions with bounded first-order derivatives and the solutions can be extended globally. $\square$

#### Existence of steady states

Let the upper asterisk denote the values of the variables at a steady state. Direct calculations of steady states of (1) lead to the following dependencies

5 $$\begin{aligned} V_i^* = \frac{1}{\varepsilon _i} \left( \alpha _i S_i^* + \frac{\alpha _i \gamma _i}{\varepsilon _i + \gamma _i}I_i^* \right) \qquad \textrm{and} \qquad W_i^* = \frac{\alpha _i I_i^*}{\varepsilon _i + \gamma _i}. \end{aligned}$$

Moreover, we have

6 $$\begin{aligned} H_i^* = \frac{\varepsilon _i}{\alpha _i} C_i^* = \frac{\varepsilon _i}{\alpha _i + \varepsilon _i} G_i > 0, \end{aligned}$$

since $H_i^* + C_i^* = G_i > 0.$ Thus, the disease-free steady state of (1) is given by

7 $$\begin{aligned} E_0= & {} \left( S_1^0,S_2^0,I_1^0,I_2^0,V_1^0,V_2^0,W_1^0,W_2^0\right) \nonumber \\= & {} \left( \frac{\varepsilon _1 (1 - G_2)}{\alpha _1 + \varepsilon _1}, \frac{\varepsilon _2 G_2}{\alpha _2 + \varepsilon _2}, 0, 0, \frac{\alpha _1 (1 - G_2)}{\alpha _1 + \varepsilon _1}, \frac{\alpha _2 G_2}{\alpha _2 + \varepsilon _2}, 0, 0 \right) \end{aligned}$$

and it exists for all values of the parameters.

Let us consider steady states of (1), for which $I_1^{*},I_2^*>0$. Using $W_i^*$ from (5) and $\frac{\mathop {}\!\textrm{d}I_i^*}{\mathop {}\!\textrm{d}t} = 0$ in (1b), we get

$$\begin{aligned} S_i^* = \left( I^* + S^* \right) \frac{1}{\beta _i} \left( \alpha _i + \gamma _i - \frac{(1 - \sigma )\varepsilon _i \alpha _i}{\varepsilon _i + \gamma _i} \right) \frac{I_i^*}{I_1^* + I_2^*}, \end{aligned}$$

which is equivalent to

8 $$\begin{aligned} S_i^* = \left( H_1^* + H_2^* \right) \frac{\alpha _i + \gamma _i}{\beta _i} \left( 1 - q_i \right) \frac{I_i^*}{I_1^* + I_2^*}, \end{aligned}$$

where

9 $$\begin{aligned} q_i = \frac{(1 - \sigma )\varepsilon _i \alpha _i}{\left( \varepsilon _i + \gamma _i\right) \left( \alpha _i + \gamma _i\right) }, \qquad 0 \le q_i < 1 \end{aligned}$$

describes the probability that an individual from the *i*-th group, colonised on the discharge, remains colonised on the readmission.

Furthermore, we denote

10 $$\begin{aligned} \psi _i:= \frac{\beta _i}{\alpha _i + \gamma _i} \left( \frac{1}{1 - q_i} \right) > 0. \end{aligned}$$

Parameter $\psi _i$ is a product of two factors. The first one, $\frac{\beta _i}{\alpha _i + \gamma _i}$, indicates the number of secondary cases one infectious individual would cause during their stay in the hospital, if all individuals belonged to the *i*-th group. The second factor describes the sum of probabilities that a patient from that group remains colonised upon any subsequent admission to the hospital. Parameter $\psi _i$ is simply a basic reproduction number for a model describing a single risk group *i*, which is equivalent to the model presented in^17^.

Since $S_i = H_i - I_i,$ from (8) we derive

11 $$\begin{aligned} I_1^*&= H_1^* - \left( H_1^* + H_2^*\right) \frac{1}{\psi _1}\frac{I_1^*}{I_1^* + I_2^*}, \end{aligned}$$

12 $$\begin{aligned} I_2^*&= H_2^* - \left( H_1^* + H_2^*\right) \frac{1}{\psi _2}\frac{I_2^*}{I_1^* + I_2^*}. \end{aligned}$$

In order to find explicit formulae for $I_1^*, I_2^*,$ we transform (11)–(12), obtaining

13 $$\begin{aligned} 0&= A\left( I_1^*\right) ^2 + BI_1^* + C, \end{aligned}$$

14 $$\begin{aligned} I_2^*&= -I_1^* + \frac{I_1^* r_1}{H_1^* - I_1^*}, \end{aligned}$$

where

15 $$\begin{aligned} r_i:= \frac{H_1^* + H_2^*}{\psi _i} > 0 \qquad \text {for} \qquad i=1,2 \end{aligned}$$

and

$$\begin{aligned} A=1 - \frac{r_2}{r_1}, \qquad B=r_1 - H_1^* - r_2 + H_2^* + 2 H_1^* \frac{r_2}{r_1}, \qquad C=H_1^* \left( r_2 - H_2^* - \frac{r_2}{r_1}H_1^* \right) . \end{aligned}$$

Clearly,

$$\begin{aligned} \psi _2> \psi _1 \iff r_1> r_2> 0 \iff 1 - \frac{r_2}{r_1} > 0. \end{aligned}$$

##### Theorem 2

The following statements are true:

1. System (1) always has a single disease-free steady state ($E_0$).
2. System (1) has a single endemic steady state ($E_*$) if and only if model parameters satisfy condition
  16 $$\begin{aligned} \frac{H_1^* \psi _1 + H_2^* \psi _2}{H_1^* + H_2^*} > 1, \end{aligned}$$
  where $H_i^*$ and $\psi _i$ are given by (6) and (10), respectively.

##### Proof

The existence of the disease-free steady state is obvious. By (14), we find that

$$\begin{aligned} I_2^*> 0 \iff 0< I_1^* < \frac{I_1^* r_1}{H_1^* - I_1^*} \iff r_1> H_1^* - I_1^* > 0. \end{aligned}$$

Thus, any non-negative endemic steady state of (1) is given by real solutions $I_1^*$ to Eq. (13) satisfying

17 $$\begin{aligned} H_1^*> I_1^* > \max (0, H_1^* - r_1). \end{aligned}$$

If $\psi _2 > \psi _1$ (i.e. $A>0$), then Eq. (13) is quadratic. We find that $A>0$ and $C \ge 0$ implies $B > 0.$ Hence, by Vieta’s formulae, Eq. (13) has a single positive real solution if and only if $C < 0.$ Similarly, if $\psi _2 = \psi _1$, then we get $B > 0$ and the positive solution to linear Eq. (13) exists only for $C < 0.$ In both cases, it can be easily shown that such solutions satisfy condition (17).

Since $H_i^* > 0,$ inserting formula (15) into condition $C < 0$ yields

$$\begin{aligned} r_2< H_2^* + \frac{r_2}{r_1} H_1^* \iff \frac{1}{\psi _2} \left( H_1^* + H_2^*\right) < H_2^* + \frac{\psi _1}{\psi _2} H_1^* \iff \frac{H_1^* \psi _1 + H_2^* \psi _2}{H_1^* + H_2^*} > 1. \end{aligned}$$

On the other hand, when $\psi _1 > \psi _2$, due to the symmetry of a problem, it is sufficient to consider polynomial analogous to (13), but with respect to $I_2^*$ instead. It would be equivalent to swapping indices and the considered equation would also have a single positive real solution, which would satisfy condition analogous to (17).

Concluding, if (16) holds, then system (1) has a single endemic steady state. Otherwise, it has no non-negative steady states other than the disease-free steady state $x_0.$ $\square$

##### Remark 3

Theorem 2 illustrates the impact of the introduction of two risk groups interacting with each other. If these groups were separate, the endemic state would be present independently in these groups, provided that $\psi _i > 1$ for the given group (using results of^17^). If both groups are present in the same hospital and they interact with each other, their individual basic reproduction numbers $\psi _i$ lose their previous interpretations. Instead, the endemic state of the system depends on the sum of these numbers with weights, i.e. $H_i^* \psi _i / \left( H_1^* + H_2^*\right)$. These weights are simply proportions of the given group population to the total population of the hospital. From the proof, we also conclude that there cannot be an endemic state within one group, with a disease-free state in the other group, as either $I_1^* = I_2^* = 0$, or $I_1^* > 0$ and $I_2^* > 0$.

#### Basic reproduction number and stability of steady states

To analyse the local stability of steady states of system (1), we use the next generation matrix approach proposed by Diekmann et al.^27^ and van den Driessche and Watmough^28^ and derive the formula for the so-called basic reproduction number $\mathscr {R}_0$.

Using $V_i = G_i - S_i - I_i - W_i,$ we reduce and rewrite system (1) as

18 $$\begin{aligned} \frac{\textrm{d}x}{\textrm{d}t} = f(x) = {\mathscr {F}}(x)-{\mathscr {V}}(x)= {\mathscr {F}}(x)-({\mathscr {V}^-}(x)- {\mathscr {V}^+}(x)), \end{aligned}$$

where

$$\begin{aligned} x=(x_1,x_2,x_3,x_4,x_5,x_6):=(I_1,I_2,W_1,W_2,S_1,S_2) \end{aligned}$$

and

19 $$\begin{aligned} \mathscr {F}(x)= & {} \begin{pmatrix} \beta _1 \frac{x_1+x_2}{x_1+x_2+x_5+x_6} x_5 \\ \beta _2 \frac{x_1+x_2}{x_1+x_2+x_5+x_6} x_6 \\ 0 \\ 0 \\ 0 \\ 0 \end{pmatrix}; \quad \mathscr {V}^-(x) = \begin{pmatrix} (\alpha _1 + \gamma _1) x_1 \\ (\alpha _2 + \gamma _2) x_2 \\ (\varepsilon _1 + \gamma _1) x_3 \\ (\varepsilon _2 + \gamma _2) x_4 \\ \beta _1 \frac{x_1+x_2}{x_1+x_2+x_5+x_6} x_5 + \alpha _1 x_5 \\ \beta _2 \frac{x_1+x_2}{x_1+x_2+x_5+x_6} x_6 + \alpha _2 x_6 \\ \end{pmatrix}; \nonumber \\ \mathscr {V}^+(x)= & {} \begin{pmatrix} (1 - \sigma ) \varepsilon _1 x_3 \\ (1 - \sigma ) \varepsilon _2 x_4 \\ \alpha _1 x_1 \\ \alpha _2 x_2 \\ \gamma _1 x_1 + \varepsilon _1 (G_1 - x_5 - x_1 - x_3) + \sigma \varepsilon _1 x_3 \\ \gamma _2 x_2 + \varepsilon _2 (G_2 - x_6 - x_2 - x_4) + \sigma \varepsilon _2 x_4 \\ \end{pmatrix}. \end{aligned}$$

The dynamics of colonised patient populations, i.e. $I_1, I_2, W_1, W_2,$ are described by the first four equations. Functions defined in (19) are interpreted as follows: $\mathscr {F}_i(x)$ is the rate of appearance of new infections in compartment *i*, $\mathscr {V}^+_i(x)$ is the rate of transfer of individuals into compartment *i* by all other means, and $\mathscr {V}^-_i(x)$ is the rate of transfer of individuals out of compartment *i*.

Let us define

20 $$\begin{aligned} F(x_0) = \left[ \frac{\partial \mathscr {F}_i}{\partial x_j}(x_0) \right] \qquad \text {and} \qquad V(x_0) = \left[ \frac{\partial \mathscr {V}_i}{\partial x_j}(x_0) \right] \quad \text {for} \quad i,j = 1, 2, \dots , 4, \end{aligned}$$

where disease-free steady state reads

21 $$\begin{aligned} x_0 = \left( 0, 0, 0, 0,\frac{\varepsilon _1 (1 - G_2)}{\alpha _1 + \varepsilon _1}, \frac{\varepsilon _2 G_2}{\alpha _2 + \varepsilon _2}\right) . \end{aligned}$$

The next generation matrix of system (1) is given by $FV^{-1}(x_0),$ while the basic reproduction number $\mathscr {R}_0$ — by the spectral radius of this matrix.

For system (18), matrices *F* and *V* have the following form:

$$\begin{aligned} F(x_0) = \begin{bmatrix} d_{1}^{I}(x_0) &{} d_{1}^{I}(x_0) &{} 0 &{} 0 \\ d_{2}^{I}(x_0) &{} d_{2}^{I}(x_0) &{} 0 &{} 0 \\ 0 &{} 0 &{} 0 &{} 0 \\ 0 &{} 0 &{} 0 &{} 0 \end{bmatrix}, \qquad V(x_0) = \begin{bmatrix} \alpha _1 + \gamma _1 &{} 0 &{} (\sigma -1)\varepsilon _1 &{} 0 \\ 0 &{} \alpha _2 + \gamma _2 &{} 0 &{} (\sigma -1)\varepsilon _2 \\ -\alpha _1 &{} 0 &{} \varepsilon _1 + \gamma _1 &{} 0 \\ 0 &{} -\alpha _2 &{} 0 &{} \varepsilon _2 + \gamma _2 \end{bmatrix}, \end{aligned}$$

where

22 $$\begin{aligned} d_{i}^{I}(x_0)= & {} \frac{\partial }{\partial x_{j}} \left( \beta _i \frac{x_1 + x_2}{x_1 + x_2 + x_5 + x_6} x_{4+i} \right) \Bigg |_{x=x_0}\nonumber \\= & {} \beta _i \left( \frac{H_i^*}{H_1^* + H_2^*} \right) , \quad i,j=1,2. \end{aligned}$$

Direct calculations of the next generation matrix yield

$$\begin{aligned} FV^{-1}(x_0) = \begin{bmatrix} \frac{d_{1}^{I} (x_0)}{\det V_1}(\varepsilon _1 + \gamma _1) &{} \frac{d_{1}^{I} (x_0)}{\det V_2}(\varepsilon _2 + \gamma _2) &{} \frac{d_{1}^{I} (x_0)}{\det V_1}(1 - \sigma )\varepsilon _1 &{} \frac{d_{1}^{I} (x_0)}{\det V_2}(1 - \sigma )\varepsilon _2 \\ \frac{d_{2}^{I} (x_0)}{\det V_1}(\varepsilon _1 + \gamma _1) &{} \frac{d_{2}^{I} (x_0)}{\det V_2}(\varepsilon _2 + \gamma _2) &{} \frac{d_{2}^{I} (x_0)}{\det V_1}(1 - \sigma )\varepsilon _1 &{} \frac{d_{2}^{I} (x_0)}{\det V_2}(1 - \sigma )\varepsilon _2 \\ 0 &{} 0 &{} 0 &{} 0 \\ 0 &{} 0 &{} 0 &{} 0 \end{bmatrix}, \end{aligned}$$

where

$$\begin{aligned} \det V_i = \left( \alpha _i + \gamma _i \right) \left( \varepsilon _i + \gamma _i \right) \left( 1 - q_i \right) , \qquad i = 1, 2. \end{aligned}$$

Thus, $FV^{-1}(x_0)$ has only one non-zero eigenvalue and we have

23 $$\begin{aligned} \mathscr {R}_0= & {} \sum _{1=1}^2\frac{d_{i}^{I} (x_0)}{\det V_i}(\varepsilon _i + \gamma _i)\nonumber \\= & {} \frac{1}{H_1^* + H_2^*}\left( \frac{H_1^* \beta _1}{\left( \alpha _1 + \gamma _1 \right) \left( 1 - q_1 \right) } + \frac{H_2^* \beta _2}{\left( \alpha _2 + \gamma _2 \right) \left( 1 - q_2 \right) }\right) \nonumber \\= & {} \frac{H_1^* \psi _1 + H_2^* \psi _2}{H_1^* + H_2^*}, \end{aligned}$$

where $H_i^*$ and $\psi _i$ are given by (6) and (10), respectively. Clearly, $\mathscr {R}_0$ is a weighted arithmetic mean of the reproduction numbers for the model of disjoint risk groups (10) with weights given by (6) (see also Remark 3).

Let us recall that if $\mathscr {R}_0 > 1,$ then by Theorem 2 system (1) has an endemic steady state.

##### Theorem 4

Consider $\mathscr {R}_0$ and $E_0$ given by (23) and (7), respectively. Then

1. For $\mathscr {R}_0<1$ system (1) has exactly one non-negative globally asymptotically stable steady state $E_0$ (called the disease-free);
2. For $\mathscr {R}_0>1$ system (1) has two non-negative steady states: $E_0$, which is unstable, and endemic steady state $E_*$, which is globally asymptotically stable.
3. For $\mathscr {R}_0=1$ we observe a forward bifurcation for system (1).

##### Proof

Since system (18) satisfies conditions (A1)–(A5) postulated in^28^, disease-free steady state $E_0$ is locally asymptotically stable for $\mathscr {R}_0<1$ and unstable for $\mathscr {R}_0>1$ due to Theorem 2 in^28^.

Let us rewrite system (1) into an equivalent form

24a $$\begin{aligned} \frac{\mathop {}\!\textrm{d}H_i}{\mathop {}\!\textrm{d}t}&= - \alpha _i H_i + \varepsilon _i \left( G_i-H_i\right) , \end{aligned}$$

24b $$\begin{aligned} \frac{\mathop {}\!\textrm{d}I_i}{\mathop {}\!\textrm{d}t}&= \beta _i \frac{I_1+I_2}{H_1+H_2} \left( H_i-I_i\right) - \alpha _i I_i - \gamma _i I_i + (1 - \sigma ) \varepsilon _i W_i ,\nonumber \\ \frac{\mathop {}\!\textrm{d}W_i}{\mathop {}\!\textrm{d}t}&= \alpha _i I_i - \varepsilon _i W_i - \gamma _i W_i, \end{aligned}$$

for $i=1,2$. System (24a, 24b) consists of two cascading subsystems (24a) and (24b). Subsystem (24a) comprises 2 equations and describes the changes in the total populations of given risk groups in the hospital, while subsystem (24b) includes remaining 4 equations and describes the changes in the colonised populations in both hospital and community. Clearly, solutions of subsystem (24a) do not depend on the variables described by subsystem (24b).

First, consider subsystem (24a). From (3) it follows that this subsystem has a globally asymptotically stable steady state given by

25 $$\begin{aligned} H_1^*=\frac{\varepsilon _1}{\alpha _1+\varepsilon _1}G_1,\quad H_2^*=\frac{\varepsilon _2}{\alpha _2+\varepsilon _2}G_2. \end{aligned}$$

Next, consider subsystem (24b) at the equilibrium of system (24a), namely

26 $$\begin{aligned} \frac{\mathop {}\!\textrm{d}I_i}{\mathop {}\!\textrm{d}t}= & {} \beta _i \frac{I_1+I_2}{H_1^*+H_2^*} \left( H_i^*-I_i\right) - \alpha _i I_i - \gamma _i I_i + (1 - \sigma ) \varepsilon _i W_i,\nonumber \\ \frac{\mathop {}\!\textrm{d}W_i}{\mathop {}\!\textrm{d}t}= & {} \alpha _i I_i - \varepsilon _i W_i - \gamma _i W_i, \end{aligned}$$

for $i=1,2$. Note that 4-dimensional set $K=\{0\le I_i\le H_i^*$, $0 \le W_i\le C_i^*, i=1,2\}$ is positively invariant with respect to (26). We prove the non-negativity of the variables using the same method as in the proof of Statement 1. Similarly, let $t_0$ denote the first time any of the variables $I_1$, $I_2$, $W_1$, $W_2$ reaches its upper bound in set *K*. By (6), for $W_i(t_0)=C_i^*$ we have

$$\begin{aligned} \begin{aligned} \frac{\mathop {}\!\textrm{d}W_i(t_0)}{\mathop {}\!\textrm{d}t}&= \alpha _i I_i(t_0)-\left( \varepsilon _i+\gamma _i\right) C_i^*\le \alpha _i H_i^*-\left( \varepsilon _i+\gamma _i\right) C_i^*\\&= \varepsilon _i C_i^*-\left( \varepsilon _i+\gamma _i\right) C_i^*<0, \end{aligned} \end{aligned}$$

and for $I_i(t_0)=H_i^*$ we have

$$\begin{aligned} \begin{aligned} \frac{\mathop {}\!\textrm{d}I_i(t_0)}{\mathop {}\!\textrm{d}t}&= -(\alpha _i+\gamma _i)H_i^*+(1-\sigma )\varepsilon _i W_i(t_0)\le -(\alpha _i+\gamma _i)H_i^*+\varepsilon _i C_i^*\\&= -(\alpha _i+\gamma _i)H_i^*+\alpha _i H_i^*<0. \end{aligned} \end{aligned}$$

Furthermore, since system (26) is cooperative and its Jacobian matrix is irreducible in the interior of *K*, it generates a monotone flow on *K* and a strongly monotone flow on interior of *K*, giving a strongly monotone flow on *K* as a result (^29^, Theorem 1.7). It is easy to verify that the Jacobian matrix of system (26) is also antimonotone. By^29^, Theorem 6.1 restricted to the set *K* instead of $\mathbb {R}^{n}_{+}$, we obtain that either all solutions tend to 0 (corresponding to the case $\mathscr {R}_0<1$), or they tend to a unique steady state $\left( I_1^*, I_2^*, W_1^*, W_2^*\right)$ with all coordinates positive (corresponding to the case $\mathscr {R}_0>1$).

If $\mathcal{R}_0<1$, then the attractor 0 of system (26) is stable by Theorem 2 in^28^. On the other hand, if $\mathcal{R}_0>1$, then the Jacobian matrix evaluated at $(I_1^*, I_2^*, W_1^*, W_2^*)$ is a Metzler matrix that is element-wise less than

$$\begin{aligned}\begin{bmatrix} \frac {\beta_1 (H_1^*-I_1^*)}{H_1^*+H_2^*}-\alpha_1-\gamma_1 & \frac {\beta_1 (H_1^*-I_1^*)}{H_1^*+H_2^*} &{} (1 - \sigma ) \varepsilon _1 &{} 0 \\ \frac {\beta_2 (H_2^*-I_2^*)}{H_1^*+H_2^*} &{} \frac {\beta_2 (H_2^*-I_2^*)}{H_1^*+H_2^*} - \alpha _2 - \gamma _2 &{} 0 &{} (1 - \sigma ) \varepsilon _2 \\ \alpha _1 &{} 0 &{} -\varepsilon _1 - \gamma _1 &{} 0 \\ 0 &{} \alpha _2 &{} 0 &{} -\varepsilon _2 - \gamma _2 \end{bmatrix}. \end{aligned}$$

Positive vector $(I_1^*, I_2^*, W_1^*, W_2^*)$ belongs to the kernel of matrix (Metzler matrix), so, by Perron-Frobenius Theorem, 0 is a simple eigenvalue of (Metzler matrix) and the remaining eigenvalues have negative real parts. Furthermore, there exists $a>0$ such that after adding the matrix $a\cdot\mathrm{Id}$, both Jacobian matrix evaluated at $(I_1^*, I_2^*, W_1^*, W_2^*)$ and matrix (Metzler matrix) are non-negative and irreducible. By Corollary 2.1.5^30^, all eigenvalues of the Jacobian matrix evaluated at $(I_1^*, I_2^*, W_1^*, W_2^*)$ have negative real parts. Thus, for $\mathcal{R}_0>1$ steady state $(I_1^*, I_2^*, W_1^*, W_2^*)$ of system (26) is stable.

Let us define a point *P* as a combination of stable steady states of subsystems (24a) and (26)

$$\begin{aligned} P={\left\{ \begin{array}{ll} (H_1^*, H_2^*, 0, 0, 0, 0)\quad &{}\text { if }\mathscr {R}_0<1,\\ (H_1^*, H_2^*, I_1^*, I_2^*, W_1^*, W_2^*)\quad &{}\text { if }\mathscr {R}_0>1. \end{array}\right. } \end{aligned}$$

For $\mathscr {R}_0<1$ the point *P* corresponds to the disease-free steady state $E_0$ given by (7), while for $\mathscr {R}_0>1$ it corresponds to the endemic steady state $E_*$, where values $I_i^*$, $W_i^*$ satisfy (5), (13) and (14). In order to prove the global stability of *P* as a steady state of (24), we use arguments inspired by proof of^31^, Theorem 4.2.

First, by^32^, Theorem 2 or^33^, Theorem 3.1 *P* is locally asymptotically stable steady state of (24). Thus, we only need to prove the global attractivity of the point *P*.

Consider a trajectory of system (24) starting at any point satisfying the initial conditions (2).

The non-negativity of solutions (see Statement 1) indicates that, for all positive *t* the following conditions are satisfied

$$\begin{aligned} 0\le I_i(t)\le H_i(t)\quad \text { and }\quad 0\le W_i(t)\le C_i(t),\quad i=1,2. \end{aligned}$$

Since $(H_1^*,H_2^*)$ is a globally asymptotically stable steady state of (24a), for every $\varepsilon >0$ there exists $T>0$ such that after time *T* the considered trajectory of system (24) is contained in set

$$\begin{aligned} \left\{ |H_i-H_i^*|\le \varepsilon , \ 0\le I_i\le H_i^*+\varepsilon ,\ 0 \le W_i(0)\le C_i^*+\varepsilon ,\ i=1,2\right\} . \end{aligned}$$

Therefore, the $\omega$-limit set of this trajectory is a subset of

$$\begin{aligned} \{H_1^*\}\times \{H_2^*\}\times [0,H_1^*]\times [0,H_2^*]\times [0,C_1^*]\times [0,C_2^*]. \end{aligned}$$

Assume that in the $\omega$-limit set of the considered trajectory there is a point $P_1=(H_1^*,H_2^*,i_1^*,i_2^*,w_1^*,w_2^*)\ne P$. Choose $\varepsilon >0$ such that $\varepsilon <||P-P_1||$. From the local asymptotic stability of *P* there exists such $\delta >0$ that solutions starting at any point in $\delta$-neighbourhood of *P* do not leave $\varepsilon$-neighbourhood of *P*.

Observe that the last four variables of the solution to system (24) with initial point $P_1$ are equal to the solution of system (26) with an initial point $(i_1^*,i_2^*,w_1^*,w_2^*)$. Since $i_i^*\in [0,H_i^*]$ and $w_i^*\in [0,C_i^*]$ for $i=1,2$, we obtain that the trajectory starting at $P_1$ converges to *P*. In particular, for any $0<\eta <\delta$ there exists $T>0$ such that after time *T* solution starting at $P_1$ does not leave $\eta$-neighbourhood of *P*.

From the continuity of solutions with respect to initial conditions, there exists a small enough neighbourhood of $P_1$ such that after time *T* all the trajectories starting at this neighbourhood belong to $\delta$-neighbourhood of *P* and, thus, these trajectories do not leave the $\varepsilon$-neighbourhood of *P*. Hence, $P_1$ cannot belong to the $\omega$-limit set of the initially chosen trajectory and *P* is the only element in this $\omega$-limit set.

Since system (24) and system (1) are equivalent, for $\mathscr {R}_0<1$ the state $E_0$ is globally asymptotically stable and for $\mathscr {R}_0>1$ the endemic steady state $E_*$ is globally asymptotically stable. As a consequence, for $\mathscr {R}_0=1$ we observe a forward bifurcation. Alternatively, to investigate the type of the bifurcation at $\mathscr {R}_0=1$ one can follow the approach proposed by van den Driessche and Watmough and check the assumptions of Theorem 4 from^28^; for details see Statement 5 and its proof. $\square$

##### Statement 5

The following statements are true:

1. For $\mathscr {R}_0<1$ system (18) has exactly one non-negative locally asymptotically stable steady state $x_0$ (called the disease-free);
2. For $\mathscr {R}_0>1$ system (18) has two non-negative steady states: $x_0$, which is unstable, and endemic steady state $x_*$. Moreover, there exists $\varepsilon >0$ such that $x_*$ is locally asymptotically stable for $\mathscr {R}_0$ satisfying $1+\varepsilon>\mathscr {R}_0>1$;
3. For $\mathscr {R}_0=1$ we observe a forward bifurcation for system (18),

where $\mathscr {R}_0$ and $x_0$ are given by (23) and (21), respectively.

##### Proof

Since system (18) satisfies conditions (A1)-(A5) postulated in^28^, the statements regarding the stability of disease-free steady state $x_0$ are the direct consequences of Theorem 2 in^28^. Namely, $x_0$ is locally asymptotically stable for $\mathscr {R}_0<1$ and unstable for $\mathscr {R}_0>1$.

In order to investigate the stability of the endemic steady state and also the type of the bifurcation occurring at $\mathscr {R}_0 = 1,$ we use an approach based on the centre manifold theory, as proposed in^28^. Let us define a bifurcation parameter $\mu =\beta _2 - \bar{\beta },$ where

27 $$\begin{aligned} \bar{\beta } = \frac{H_1^*}{H_2^*}\left( \alpha _2 + \gamma _2 \right) \left( 1 - q_2 \right) \left( 1 - \psi _1 + \frac{H_2^*}{H_1^*} \right) . \end{aligned}$$

Clearly, for such parameter $\bar{\beta }$ we have $\mu = 0 \iff \beta _2=\bar{\beta }\iff \mathscr {R}_0 = 1,$ while

28 $$\begin{aligned} \mu> 0 \iff \beta _2>\bar{\beta }\iff \mathscr {R}_0 > 1, \qquad \text {and} \qquad \mu< 0 \iff \beta _2<\bar{\beta }\iff \mathscr {R}_0 < 1. \end{aligned}$$

Below we show that there exists a $\delta > 0$ such that there is a locally asymptotically stable endemic steady state near the disease-free steady state for $\delta>\mu >0$. To do so, we follow van den Driessche and Watmough and check the assumptions of Theorem 4 in^28^, i.e. we verify that $a<0$, $b\ne 0$, for *a* and *b* given by

29 $$\begin{aligned} a&= \frac{v}{2} D_{xx}f(x_0, \bar{\beta }) w^2 = \frac{1}{2} \sum _{i,j,k=1}^{6} v_i w_j w_k \frac{\partial ^2 f_i}{\partial x_j \partial x_k}(x_0, \bar{\beta }), \end{aligned}$$

30 $$\begin{aligned} b&= vD_{x\beta }f(x_0, \bar{\beta })w = \sum _{i,j=1}^{6} v_i w_j \frac{\partial ^2 f_i}{\partial x_j \partial \beta _2}(x_0, \bar{\beta }) . \end{aligned}$$

where *v* and *w* are left and right null-vectors of $D_xf(x_0, \bar{\beta })$. Furthermore, we check if zero is a simple eigenvalue of $D_xf(x_0, \bar{\beta })$ i.e. the Jacobian matrix of the system (18) evaluated at the point $(x_0, \bar{\beta })$. Direct calculations lead to

31 $$\begin{aligned} D_xf(x, \beta )=\begin{bmatrix} d_{1}^{I}(x) - (\alpha _1 + \gamma _1) &{} d_{1}^{I}(x) &{} (1 - \sigma ) \varepsilon _1 &{} 0 &{} d_{1}^{S}(x) &{} d_{1}^{H}(x) \\ d_{2}^{I}(x) &{} d_{2}^{I}(x) - (\alpha _2 + \gamma _2) &{} 0 &{} (1 - \sigma ) \varepsilon _2 &{} d_{2}^{H}(x) &{} d_{2}^{S}(x) \\ \alpha _1 &{} 0 &{} -\varepsilon _1 - \gamma _1 &{} 0 &{} 0 &{} 0 \\ 0 &{} \alpha _2 &{} 0 &{} -\varepsilon _2 - \gamma _2 &{} 0 &{} 0 \\ -d_{1}^{I}(x) + (\gamma _1 - \varepsilon _1) &{} -d_{1}^{I}(x) &{} (\sigma - 1) \varepsilon _1 &{} 0 &{} -d_{1}^{S}(x) - (\alpha _1 + \varepsilon _1) &{} -d_{1}^{H}(x) \\ -d_{2}^{I}(x) &{} -d_{2}^{I}(x) + (\gamma _2 - \varepsilon _2) &{} 0 &{} (\sigma - 1) \varepsilon _2 &{} -d_{2}^{H}(x) &{} -d_{2}^{S}(x) - (\alpha _2 + \varepsilon _2) \end{bmatrix}, \end{aligned}$$

where, for $$i= 1,2,$$

32 $$\begin{aligned} d_{i}^{I}(x)&= \frac{\partial }{\partial x_{j}} \left( \beta _i \frac{x_1 + x_2}{x_1 + x_2 + x_5 + x_6} x_{4+i} \right) = \beta _i \left( \frac{x_5 + x_6}{\left( x_1 + x_2 + x_5 + x_6\right) ^2} \right) x_{4+i}, \quad j=1,2, \nonumber \\ d_{i}^{S}(x)&= \frac{\partial }{\partial x_{4+i}} \left( \beta _i \frac{x_1 + x_2}{x_1 + x_2 + x_5 + x_6} x_{4+i} \right) = \beta _i \left( \frac{x_1 + x_2}{\left( x_1 + x_2 + x_5 + x_6\right) ^2} \right) (x_1 + x_2 + x_{7-i}), \end{aligned}$$

33 $$\begin{aligned} d_{i}^{H}(x)&= \frac{\partial }{\partial x_{7-i}} \left( \beta _i \frac{x_1 + x_2}{x_1 + x_2 + x_5 + x_6} x_{4+i} \right) = - \beta _i \left( \frac{x_1 + x_2}{\left( x_1 + x_2 + x_5 + x_6\right) ^2} \right) x_{4+i}. \end{aligned}$$

Clearly, for $x=x_0$ we have $d_i^S = d_i^H = 0$. Denoting $d_{i}^{I}(x_0)=d_{i}^{I},$ we get

34 $$\begin{aligned} D_xf(x_0, \bar{\beta }) = \begin{bmatrix} d_{1}^{I} - (\alpha _1 + \gamma _1) &{} d_{1}^{I} &{} (1 - \sigma ) \varepsilon _1 &{} 0 &{} 0 &{} 0 \\ d_{2}^{I} &{} d_{2}^{I} - (\alpha _2 + \gamma _2) &{} 0 &{} (1 - \sigma ) \varepsilon _2 &{} 0 &{} 0 \\ \alpha _1 &{} 0 &{} -\varepsilon _1 - \gamma _1 &{} 0 &{} 0 &{} 0 \\ 0 &{} \alpha _2 &{} 0 &{} -\varepsilon _2 - \gamma _2 &{} 0 &{} 0 \\ -d_{1}^{I} + (\gamma _1 - \varepsilon _1) &{} -d_{1}^{I} &{} (\sigma - 1) \varepsilon _1 &{} 0 &{} -\alpha _1 - \varepsilon _1 &{} 0 \\ -d_{2}^{I} &{} -d_{2}^{I} + (\gamma _2 - \varepsilon _2) &{} 0 &{} (\sigma - 1) \varepsilon _2 &{} 0 &{} -\alpha _2 - \varepsilon _2 \end{bmatrix}. \end{aligned}$$

It is clear that at least two eigenvalues of $D_xf(x_0, \bar{\beta })$ are non-zero, thus it is enough to consider $4 \times 4$ upper left block of the matrix. Calculating the coefficients of that block’s characteristic polynomial directly, it can be shown that the constant term is equal to 0,  while the linear-term coefficient is non-zero. Hence, the zero eigenvalue of $D_xf(x_0, \bar{\beta })$ is simple.

In addition, the only non-zero second-order derivatives of the right-hand side of (18) are equal to the first-order derivatives of $d^I_i, d^S_i, d^H_i$ with respect to $x_1, \dots , x_6:$

$$\begin{aligned} \frac{\partial d^{I}_i}{\partial x_1}&= \frac{\partial d^{I}_i}{\partial x_2} = -2 \beta _i \frac{x_5 + x_6}{(x_1 + x_2 + x_5 + x_6)^3} x_{4+i}, \quad&i=1,2,\\ \frac{\partial d^{S}_1}{\partial x_1}&= \frac{\partial d^{S}_1}{\partial x_2} = \frac{\partial d^{I}_1}{\partial x_5} = \beta _1 \frac{2(x_1 + x_2)x_5 + (x_1 + x_2 + x_5)x_6 + x_6^2}{(x_1 + x_2 + x_5 + x_6)^3}, \\ \frac{\partial d^{S}_2}{\partial x_1}&= \frac{\partial d^{S}_2}{\partial x_2} = \frac{\partial d^{I}_2}{\partial x_6} = \beta _2 \frac{2(x_1 + x_2)x_6 + (x_1 + x_2 + x_6)x_5 + x_5^2}{(x_1 + x_2 + x_5 + x_6)^3}, \\ \frac{\partial d^{H}_i}{\partial x_1}&= \frac{\partial d^{H}_i}{\partial x_2} = \frac{\partial d^{I}_i}{\partial x_{7-i}} = \beta _i \frac{x_1 + x_2 - x_5 - x_6}{(x_1 + x_2 + x_5 + x_6)^3}x_{4+i}, \quad&i=1,2,\\ \frac{\partial d^{S}_i}{\partial x_{7-i}}&= \frac{\partial d^{H}_i}{\partial x_{4+i}} = - \beta _i \frac{(x_1 + x_2)(x_1 + x_2 + x_{7-i} - x_{4+i})}{(x_1 + x_2 + x_5 + x_6)^3}, \quad&i=1,2, \\ \frac{\partial d^{H}_i}{\partial x_{7-i}}&= 2 \beta _i \frac{x_1 + x_2}{(x_1 + x_2 + x_5 + x_6)^3}x_{4+i}, \quad&i=1,2,\\ \frac{\partial d^{S}_i}{\partial x_{4+i}}&= - 2 \beta _i \frac{(x_1 + x_2)(x_1 + x_2 + x_{7-i})}{(x_1 + x_2 + x_5 + x_6)^3}, \quad&i=1,2. \end{aligned}$$

For $x=x_0$ we have $x_1, x_2 = 0,$ and $x_{4+i} = H_i^*$, hence evaluating those derivatives at point $(x_0, \bar{\beta })$ we get

$$\begin{aligned} \frac{\partial d^{I}_1}{\partial x_1} (x_0, \bar{\beta })&= \frac{\partial d^{I}_1}{\partial x_2} (x_0, \bar{\beta }) = -2 \beta _1 \frac{H_1^*}{(H_1^* + H_2^*)^2}, \\ \frac{\partial d^{I}_2}{\partial x_1} (x_0, \bar{\beta })&= \frac{\partial d^{I}_2}{\partial x_2} (x_0, \bar{\beta }) = -2 \bar{\beta } \frac{H_2^*}{(H_1^* + H_2^*)^2}, \\ \frac{\partial d^{S}_1}{\partial x_1} (x_0, \bar{\beta })&= \frac{\partial d^{S}_1}{\partial x_2} (x_0, \bar{\beta }) = \frac{\partial d^{I}_1}{\partial x_5} (x_0, \bar{\beta }) = \beta _1 \frac{H_2^*}{(H_1^* + H_2^*)^2}, \\ \frac{\partial d^{S}_2}{\partial x_1} (x_0, \bar{\beta })&= \frac{\partial d^{S}_2}{\partial x_2} (x_0, \bar{\beta }) = \frac{\partial d^{I}_2}{\partial x_6} (x_0, \bar{\beta }) = \bar{\beta } \frac{H_1^*}{(H_1^* + H_2^*)^2}, \\ \frac{\partial d^{H}_1}{\partial x_1} (x_0, \bar{\beta })&= \frac{\partial d^{H}_1}{\partial x_2} (x_0, \bar{\beta }) = \frac{\partial d^{I}_1}{\partial x_6} (x_0, \bar{\beta }) = - \beta _1 \frac{H_1^*}{(H_1^* + H_2^*)^2}, \\ \frac{\partial d^{H}_2}{\partial x_1} (x_0, \bar{\beta })&= \frac{\partial d^{H}_2}{\partial x_2} (x_0, \bar{\beta }) = \frac{\partial d^{I}_2}{\partial x_5} (x_0, \bar{\beta }) = - \bar{\beta } \frac{H_2^*}{(H_1^* + H_2^*)^2}, \\ \frac{\partial d^{S}_i}{\partial x_5} (x_0, \bar{\beta })&= \frac{\partial d^{S}_i}{\partial x_6} (x_0, \bar{\beta }) = \frac{\partial d^{H}_i}{\partial x_5} (x_0, \bar{\beta }) = \frac{\partial d^{H}_i}{\partial x_6} (x_0, \bar{\beta }) = 0, \qquad i=1,2. \end{aligned}$$

Lemma 3 in^28^ indicates that $v_5 = v_6 = 0$. Thus, formula (29) for *a* can be rewritten as

$$\begin{aligned} a = \frac{1}{2} \sum _{i,j,k=1}^{6} v_i w_j w_k \frac{\partial ^2 f_i}{\partial x_j \partial x_k}(x_0, \bar{\beta }) = \frac{1}{2} (v_1 a_1 + v_2 a_2 - v_5 a_1 - v_6 a_2) = \frac{1}{2} (v_1 a_1 + v_2 a_2), \end{aligned}$$

where

$$\begin{aligned} a_i = \sum _{j=1}^{6} w_j \left( \left( w_1 + w_2 \right) \frac{\partial d^{I}_i}{\partial x_j}(x_0, \bar{\beta }) + w_{4+i} \frac{\partial d^{S}_i}{\partial x_j}(x_0, \bar{\beta }) + w_{7-i} \frac{\partial d^{H}_i}{\partial x_j}(x_0, \bar{\beta }) \right) \end{aligned}$$

Having calculated the required derivatives at $(x_0, \bar{\beta }),$ we can factor out $(H_1^* + H_2^*)^{-2}$ and write down the terms of $a_1$:

$$\begin{aligned}&j=1: \quad w_1 \beta _1 \left( -2 \left( w_1 + w_2 \right) H_1^* + w_5 H_2^* - w_6 H_1^* \right) , \\&j=2: \quad w_2 \beta _1 \left( -2 \left( w_1 + w_2 \right) H_1^* + w_5 H_2^* - w_6 H_1^* \right) , \\&j=5: \quad w_5 \beta _1 \left( w_1 + w_2 \right) H_2^*, \\&j=6: \quad -w_6 \beta _1 \left( w_1 + w_2 \right) H_1^*. \end{aligned}$$

Analogously, for $a_2$ we obtain

$$\begin{aligned}&j=1: \quad w_1 \bar{\beta } \left( -2 \left( w_1 + w_2 \right) H_2^* - w_5 H_2^* + w_6 H_1^* \right) , \\&j=2: \quad w_2 \bar{\beta } \left( -2 \left( w_1 + w_2 \right) H_2^* - w_5 H_2^* + w_6 H_1^* \right) , \\&j=5: \quad -w_5 \bar{\beta } \left( w_1 + w_2 \right) H_2^*, \\&j=6: \quad w_6 \bar{\beta } \left( w_1 + w_2 \right) H_1^*. \end{aligned}$$

Direct calculations of right eigenvector of $D_xf(x_0, \bar{\beta })$ show that $w_1 = -w_5$ and $w_2 = -w_6,$ implying that

$$\begin{aligned} a_1&= - \frac{2 \left( w_1 + w_2 \right) \beta _1}{(H_1^* + H_2^*)^2} \left( w_1 H_2^* + w_1 H_1^* \right) = - \frac{2 \left( w_1 + w_2 \right) \beta _1}{H_1^* + H_2^*} w_1, \\ a_2&= - \frac{2 \left( w_1 + w_2 \right) \bar{\beta }}{(H_1^* + H_2^*)^2} \left( w_2 H_1^* + w_2 H_2^* \right) = - \frac{2 \left( w_1 + w_2 \right) \bar{\beta }}{H_1^* + H_2^*} w_2, \end{aligned}$$

and as a consequence we have

35 $$\begin{aligned} a = - \frac{w_1 + w_2}{H_1^* + H_2^*} \left( \beta _1 v_1 w_1 + \bar{\beta } v_2 w_2 \right) . \end{aligned}$$

Clearly if $v_1, v_2, w_1, w_2 > 0,$ then the expression (35) is negative. From Lemma 3 of^28^ we have $v_1, v_2, w_1, w_2 \ge 0.$ Moreover, direct calculations show that $v_1 w_1, v_2 w_2 > 0,$ yielding $a < 0.$ On the other hand, we find that the only second-order derivatives that appear in (30) that are non-zero are

$$\begin{aligned} \frac{\partial ^2 f_{2}}{\partial x_{1}\partial \beta _2} (x, \beta ) = \frac{\partial ^2 f_{2}}{\partial x_{2}\partial \beta _2} (x, \beta ) = - \frac{\partial ^2 f_{6}}{\partial x_{1}\partial \beta _2} (x, \beta ) = - \frac{\partial ^2 f_{6}}{\partial x_{2}\partial \beta _2} (x, \beta )&= \frac{d^{I}_2 \left( x \right) }{\beta _2}, \\ \frac{\partial ^2 f_{2}}{\partial x_{5}\partial \beta _2} (x, \beta ) = - \frac{\partial ^2 f_{6}}{\partial x_{5}\partial \beta _2} (x, \beta )&= \frac{d^{H}_2 \left( x \right) }{\beta _2}, \\ \frac{\partial ^2 f_{2}}{\partial x_{6}\partial \beta _2} (x, \beta ) = - \frac{\partial ^2 f_{6}}{\partial x_{6}\partial \beta _2} (x, \beta )&= \frac{d^{S}_2 \left( x \right) }{\beta _2}. \end{aligned}$$

However, evaluating them at $(x_0, \bar{\beta })$ gives us

$$\begin{aligned} \frac{\partial ^2 f_{2}}{\partial x_{1}\partial \beta _2} (x_0, \bar{\beta }) = \frac{\partial ^2 f_{2}}{\partial x_{2}\partial \beta _2} (x_0, \bar{\beta }) = - \frac{\partial ^2 f_{6}}{\partial x_{1}\partial \beta _2} (x_0, \bar{\beta }) = - \frac{\partial ^2 f_{6}}{\partial x_{2}\partial \beta _2} (x_0, \bar{\beta })&= \frac{d^{I}_2 \left( x_0 \right) }{\beta _2} = \frac{H_2^*}{H_1^* + H_2^*}, \\ \frac{\partial ^2 f_{2}}{\partial x_{5}\partial \beta _2} (x_0, \bar{\beta }) = - 
\frac{\partial ^2 f_{6}}{\partial x_{5}\partial \beta _2} (x_0, \bar{\beta }) = \frac{\partial ^2 f_{2}}{\partial x_{6}\partial \beta _2} (x_0, \bar{\beta }) = - \frac{\partial ^2 f_{6}}{\partial x_{6}\partial \beta _2} (x_0, \bar{\beta })&= 0. \end{aligned}$$

Thus,

$$\begin{aligned} b = \frac{H_2^*}{H_1^* + H_2^*} (v_2 w_1 + v_2 w_2 - v_6 w_1 - v_6 w_2)=\frac{H_2^*}{H_1^* + H_2^*} v_2(w_1 + w_2). \end{aligned}$$

We showed above that $v_2 > 0$ and $w_1 \ne -w_2,$ so $b \ne 0$, which completes the proof. $\square$

In conclusion, we have shown that for $\mathscr {R}_0<1$, independently of the initial conditions, all the solutions of system (18) converge to the disease-free steady state, meaning that the prevalence of colonisation, defined as the size of colonised population divided by the size of total population, fades over time. On the other hand, for $\mathscr {R}_0>1$ there is an endemic steady state, with a constant prevalence of colonisation over time. In this case, all solutions with non-zero initial colonised population converge to the endemic steady state.

## Numerical simulations

To illustrate the pathogen spread in hospital-community pairs, we perform numerical simulations using the proposed model. First, we describe the dataset used to estimate the parameters of system (1). It allows us to estimate transfer parameters, and to compute various quantities characterising each hospital-community pair *j*, such as its basic reproduction number $\mathscr {R}_0^j$ or the average percentage of high-risk patients in the hospital. Next, we investigate how the pathogen spread is influenced by particular risk groups. Finally, we illustrate the impact of interventions on the basic reproduction numbers as well as the bacteria prevalence.

### Dataset description

The dataset was provided by AOK Lower Saxony (AOK LS), a German health insurance company. It consists of all hospitalisation records of patients insured by AOK LS between January 1st, 2008 and December 31st, 2015. Each record contains the following information: patient’s anonymised ID, birth year, sex, dates of admission and discharge, medical diagnosis codes (ICD-10 codes), anonymised ID of the healthcare facility where the patient has been admitted, and the code of state where the facility is located.

The AOK LS dataset contains $5 \, 254 \, 492$ records in total, out of which $4 \, 573 \, 584$ are from the facilities located in Lower Saxony. Since we do not have representative records from the facilities located in other states (due to the low coverage), we do not consider these data in our further analysis.

According to the data, there are 223 healthcare facilities in Lower Saxony. Among these, 60 have been inactive for at least 90 consecutive days, with no ongoing hospitalisations during that time span, hence we omit records from these facilities. As a consequence, we consider 163 facilities with $4 \, 223 \, 397$ hospitalisations of $1 \, 482 \, 176$ distinct patients. However, after removing hospitalisation records from timely inactive facilities, there are 62 313 patients with no hospitalisation records at all. These patients are excluded from further analysis as they do not contribute to any characteristic of any hospital nor community. Average yearly numbers of admissions vary between facilities, from 46.75 to 16343.38 hospitalisations. Further analysis of this dataset may be found in^34^.

### Patient stratification and parameter estimation

Following the criteria proposed in^26^, we assign patients to the high-risk group if during any hospitalisation

they have been diagnosed with at least one of the following diseases (defined by ICD-10 codes):

- C00–C96 (cancer),
- E10–E14 (diabetes mellitus),
- I50 (heart failure),
- N18.3–N18.6 (chronic kidney disease, moderate or severe),
- D80–D89 (immune system disease),
- M34–M35 (systemic sclerosis and other systemic involvement of connective tissue),
- L40 (psoriasis),
- R76 (abnormal immunological findings in serum).

Otherwise, we assign them to the low-risk group. The assignment of patients to specific groups based on ICD-10 diagnoses is related to the assumption that their immune systems can be compromised, or to their longer and more frequent stays in the hospitals.

**Figure 1:**
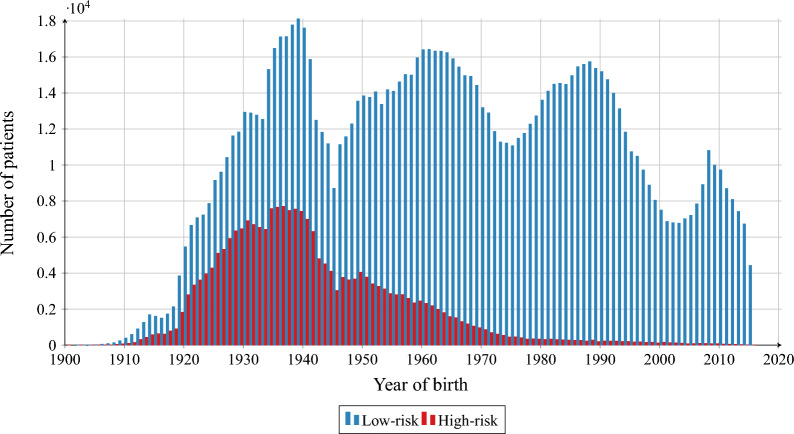
Birth year structure of the AOK LS patients hospitalised in years 2008–2015, for high-risk and low-risk groups.

**Figure 2:**
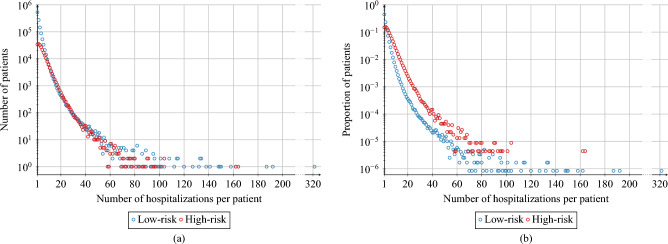
(**a**) Number of AOK LS patients with a given number of admissions/hospitalisations for high-risk and low-risk groups. (**b**) Proportion of AOK LS patients with a given number of admissions/hospitalisations for high-risk and low-risk groups with respect to the total population of the risk groups. We see that there are proportionally more high-risk patients for almost all numbers of hospitalisations greater than 2.

In the considered dataset, we identify 226 607 high-risk patients and 1 193 256 low-risk patients. High-risk patients are generally older (see Fig. 1) and on average they have 5.18 hospitalisations during the considered period, while low-risk patients have 2.55 hospitalisations. For both groups, the majority of patients (low-risk: 98.36%; high-risk: 90.08%) have not been hospitalised more than 10 times, c.f. Fig. 2.

**Figure 3:**
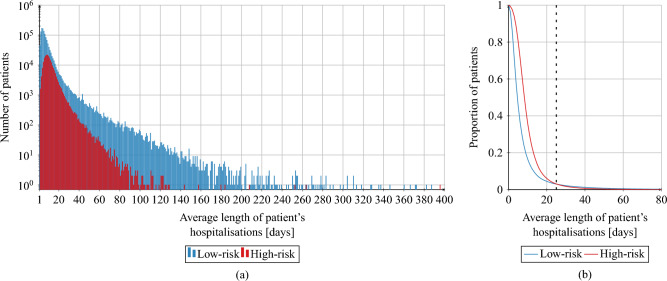
The average length of stay in a hospital for each patient from high-risk and low-risk groups in AOK LS dataset presented as a histogram (**a**) and as survival curves truncated at 80 days (**b**). For survival curves, we normalise the number of patients by dividing it by the total population of the respective risk group. We can see that for values not greater than 25 days (indicated by the dashed line) there are proportionally more low-risk patients with an average hospitalisation length shorter than the given value. However, for values greater than 25 days, the situation is the opposite. This means that low-risk patients often have either very short or very long average lengths of hospital stay.

**Figure 4:**
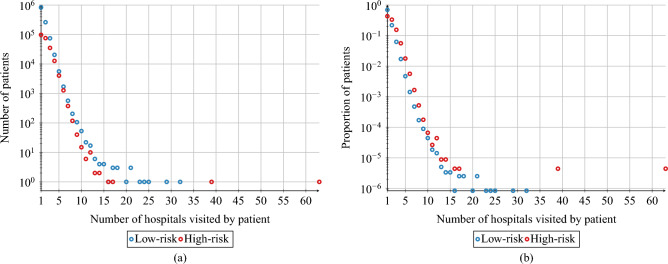
(**a**) Number of AOK LS patients with a given number of visited hospitals for high-risk and low-risk groups. (**b**) Proportion of AOK LS patients with a given number of visited hospitals for high-risk and low-risk groups with respect to the total population of the risk group.

For each patient, we compute the average length of the hospitalisation based on their records. The results are presented in Fig. 3. By taking the average of this value over the respective risk groups, we conclude that a high-risk patient spends on average 10.56 days in a hospital during a single hospitalisation, while a low-risk patient — 7.56 days. For individual patients from the low-risk group, we observe a peak in the distribution of average length of hospitalisation between days 2 and 5, while for the high-risk patients, the peak lies between days 6 and 9, as presented in Fig. 3. On average, high-risk patients visit 1.93 facilities and low-risk patients — 1.43 facilities (c.f. Fig. 4).

Using hospitalisation records, sorted by the admission date, we track the hospital and community stays of each patient. Every hospitalisation record is interpreted as a stay in a given hospital for a given number of days. The period between the date of discharge of a hospitalisation and the date of admission of the subsequent hospitalisation (including the discharge and admission dates) is interpreted as a stay in the community corresponding to the former (i.e. most recently visited) hospital. Hence, each patient is considered to stay outside of the considered hospitals and communities before their first hospitalisation record and after their last hospitalisation record.

For each hospital-community pair and each risk group, we calculate the average length of stay in the hospital and in the corresponding community and denote them as $(LOH_{i}^{j})$ and $(LOC_{i}^{j})$, respectively, where $i\in \{1,2\}$ indicates the risk group (1 – low-risk group, 2 – high-risk group) and $j\in \{1,\dots ,163\}$ – the considered hospital-community pair. Let us emphasise that we consider a system of separated hospital-community pairs rather than an interconnected network model. Thus, we do not track patient transfers between such pairs.

**Figure 5:**
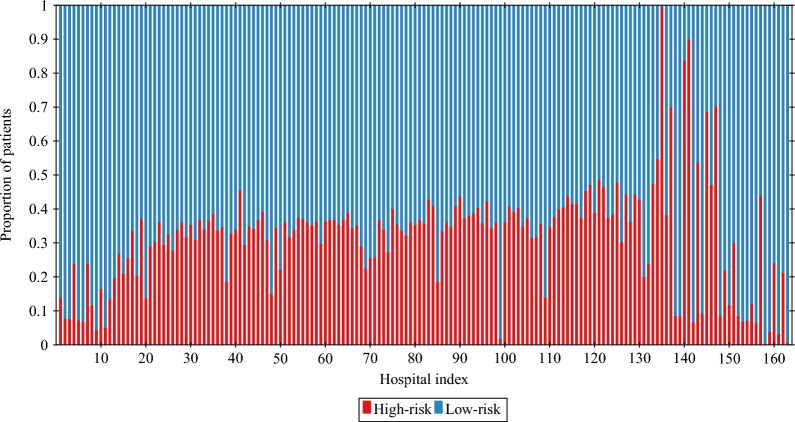
Proportion of high-risk and low-risk patients in each hospital averaged over time. Hospitals are sorted in ascending order based on $\mathscr {R}_0^j$ value, calculated using (39).

We also characterise hospital-community pairs by average pair size $PS_j$, i.e. the average daily number of patients present in the pair *j* (in either hospital *j* or community *j*), according to the previously described rules. The sum of all average pair sizes, denoted by *N*, is the total population. Furthermore, for each hospital, we compute $p_{HR}^j$, which stands for the average proportion of high-risk patients in the hospital for each day with a non-zero total hospital population. The results, presented in Fig. 5, vary substantially across different hospitals, as they range between 0.002 and 0.994, with the majority lying between 0.2 and 0.5 and the average being 0.318.

**Figure 6:**
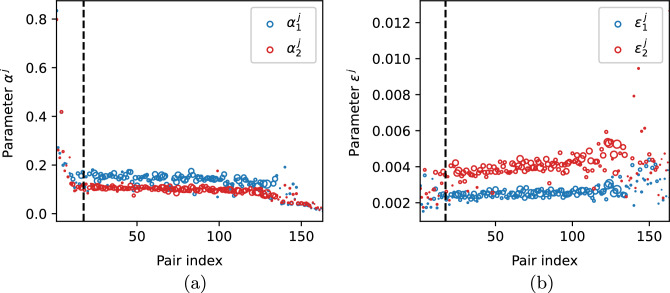
(**a**) Discharge parameters $\alpha _1^j$ and $\alpha _2^j;$ (**b**) admission parameters $\varepsilon _1^j$ and $\varepsilon _2^j$. Pairs are sorted in ascending order based on $\mathscr {R}_0^j$ value. Vertical dashed line indicates the first pair for which $\mathscr {R}_0^j > 1.$ The size of markers is proportional to the average pair size $PS_j$.

Next, we estimate the parameters of the model. Parameters $\alpha _i^j$ and $\varepsilon _i^j$ of system (1) describing the discharge and admission rates, respectively, are approximated as

$$\begin{aligned} \alpha _i^j = \frac{1}{LOH_{i}^{j}}, \quad \varepsilon _i^j = \frac{1}{LOC_{i}^{j}}, \end{aligned}$$

see Fig. 6a,b. The values of parameters $\gamma _i^j=\gamma _1=\gamma _2$ are taken from^17,18,35^ (c.f. (36)). As the base values for the transmission parameters, we take $\beta _1^j = \beta _1$ and $\beta _2^j = \beta _2 = 2 \beta _1$, since we assume that in all hospitals the transmission risk is the same and that high-risk patients are more vulnerable to susceptibility-based transmission. Moreover, $\beta _1$ is selected in such a way that the average bacteria prevalence in the community, defined as the sum of the percentages of the colonised population in the community in the endemic steady state of each hospital-community pair, multiplied by weights representing the ratio of average pair size to the total population, that is

$$\begin{aligned} \sum _{j=1}^{n} \frac{W_1^{*,j}+W_2^{*,j}}{W_1^{*,j}+W_2^{*,j}+V_1^{*,j}+V_2^{*,j}}\cdot \frac{PS_j}{N}, \end{aligned}$$

is close to $8.6\%,$ i.e. the prevalence of carriage of ESBL-producing Enterobacteriaceae in a representative sample of the general adult Dutch society

reported by Reuland et al.^36^. Thus, as a base value for the simulations, we use

36 $$\begin{aligned} \gamma _1 = \gamma _2 = 1/365 \, \text {day}^{-1} \qquad \text {and} \qquad \beta _1 = 0.0503 \, \text {day}^{-1}, \,\, \beta _2 = 0.1006 \, \text {day}^{-1}. \end{aligned}$$

The values of transmission rates $\beta _1, \beta _2$ can be additionally impacted by the interventions. In section “Prevalence of multiresistant pathogens”, we discuss two such cases. In one of them, only $\beta _2$ is affected, with its value set as low as 0.0503 day$^{-1}$. In the other case, both transmission rates are decreased by up to $30\%$ of the original values.

**Figure 7:**
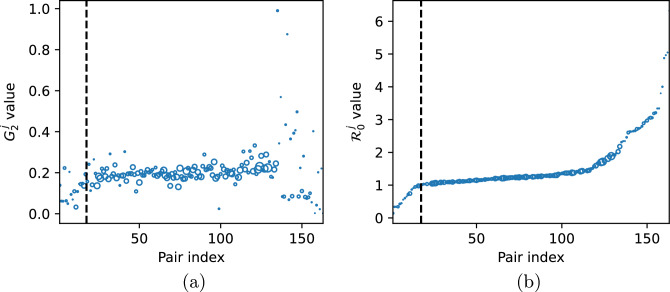
(**a**) The fraction of high-risk patients per hospital $G_2^j,$ estimated for each hospital-community pair ($j=1, \dots , 163)$; (**b**) hospital specific reproduction number $\mathscr {R}_0^j$ calculated according to formula (39). Pairs are sorted in ascending order based on $\mathscr {R}_0^j$ value. Vertical dashed line indicates the first pair for which $\mathscr {R}_0^j > 1.$ The size of markers is proportional to the average pair size $PS_j$.

Parameter values estimated from data for each hospital-community pair *j* are shown in Fig. 6. Hospitals are sorted in ascending order according to $\mathscr {R}_0^j$ value (calculated using formula (39)), c.f. Fig. 7b. For the vast majority of hospital-community pairs the lengths of stay of high-risk patients in the community between the hospitalisations ($LOC_2^j$) are shorter than for the low-risk group ($LOC_1^j$), yielding that $\varepsilon _2 > \varepsilon _1$ (159 pairs out of 163 considered). Additionally, for the majority of hospital-community pairs high-risk patients stay longer in hospitals than low-risk patients, thus $\alpha _2 < \alpha _1$ (131 pairs out of 163), see Fig. 6a,b.

### Characterisation of hospital-community pairs

For each hospital-community pair *j*, we estimate parameters $G_1^j$ and $G_2^j.$ First, we postulate the following relation between the average proportion of high-risk patients in the *j*-th hospital ($p_{HR}^j$) based on the data and theoretical populations in the endemic steady state

37 $$\begin{aligned} p_{HR}^j = \frac{H_2^{*,j}}{H_1^{*,j} + H_2^{*,j}}, \end{aligned}$$

where $H_i^{*,j}$ are fractions of individuals from the *i*-th group in *j*-th hospital in any steady state.

Clearly,

$$\begin{aligned} H_2^{*,j} \left( 1 - p_{HR}^j \right) = H_1^{*,j} \, p_{HR}^j. \end{aligned}$$

Having in mind that $G_1^j=1-G_2^j$ and plugging (6) instead of $H_i^{*, j}$ yields

38 $$\begin{aligned} \frac{\varepsilon _2^j}{\alpha _2^j + \varepsilon _2^j} G_2^j \left( 1 - p_{HR}^j \right) = \frac{\varepsilon _1^j}{\alpha _1^j + \varepsilon _1^j} \left( 1 - G_2^j \right) p_{HR}^j, \end{aligned}$$

which is equivalent to

$$\begin{aligned} \frac{G_2^j}{1 - G_2^j} = \frac{\varepsilon _1^j \left( \alpha _2^j + \varepsilon _2^j \right) p_{HR}^j}{\varepsilon _2^j \left( \alpha _1^j + \varepsilon _1^j \right) \left( 1 - p_{HR}^j \right) }. \end{aligned}$$

Hence,

$$\begin{aligned} G_2^j = \frac{x}{1 + x}, \qquad \text {where} \qquad x = \frac{\varepsilon _1^j \left( \alpha _2^j + \varepsilon _2^j \right) p_{HR}^j}{\varepsilon _2^j \left( \alpha _1^j + \varepsilon _1^j \right) \left( 1 - p_{HR}^j \right) }. \end{aligned}$$

For values of $G_2^j$ estimated from the data, see Fig. 7a. Evidently, from (23) and (37) it follows that for each hospital-community pair *j* we have

39 $$\begin{aligned} \mathscr {R}_0^j = \left( 1 - p_{HR}^j \right) \psi _1^j + p_{HR}^j \, \psi _2^j, \end{aligned}$$

where $\psi _i^j$ is an analogue of (10), easily calculated based on already estimated parameters, see Remark 3 and Fig. 7b. We found that 17 (out of 163 considered) pairs have $R_0^j<1$. However, this number depends on the transmission rates and thus it may be specific for a given pathogen and individual situation in the hospital. The latter is not taken into account in our simulations, as we assume that the transmission rates are the same in all hospitals.

In the following, we would also require the values of $H_i^{*,j}$ and $C_i^{*,j},$ which one can estimate as

40 $$\begin{aligned} H_i^{*,j} = \frac{\varepsilon _i^j}{\alpha _i^j + \varepsilon _i^j} G_i^j \qquad \text {and} \qquad C_i^{*,j}=\frac{\alpha _i^j}{\varepsilon _i^j} H_i^{*,j}, \end{aligned}$$

using previously estimated parameters.

**Figure 8:**
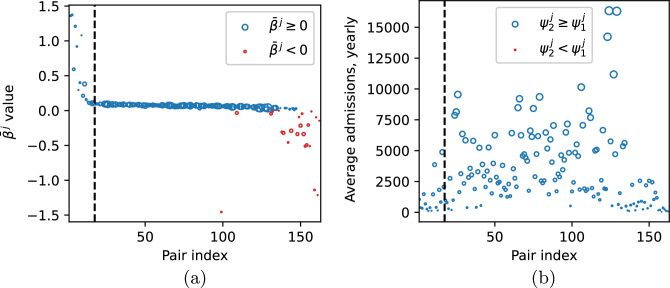
(**a**) $\bar{\beta }^j$ value evaluated for the fixed model parameters as described in Section “Patient stratification and parameter estimation” (with omitted values: 2.58, −21.48, −6.45), biologically non-feasible values marked in red; (**b**) average yearly number of admissions, for each hospital-community pair ($j = 1, \dots , 163$). There are no red markers in (**b**), as for all pairs $\psi _2^j \ge \psi _1^j.$ Pairs are sorted in ascending order based on $\mathscr {R}_0^j$ value. Vertical dashed line indicates the first pair for which $\mathscr {R}_0^j > 1.$ The size of markers is proportional to the average pair size $PS_j$.

In Fig. 8a, we report $\bar{\beta }^j$ values defined in (27) for each hospital-community pair *j*. As described in Section “Mathematical properties of the single hospital-community pair model with patient risk groups”, $\bar{\beta }^j$ is the critical value of the transmission parameter for the high-risk group for which we observe forward bifurcation in the system, assuming other parameters to be fixed. For 25 out of 163 pairs, the bifurcation occurs in the biologically non-feasible parameter region. In such cases the computed $\bar{\beta }^j$ is negative, which follows from the fact that in these cases we have

$$\begin{aligned} 1 - \psi _1^j + \frac{H_2^{*, j}}{H_1^{*, j}} < 0 \iff \psi _1^j > \frac{H_1^{*, j} + H_2^{*, j}}{H_1^{*, j}} = \frac{1}{1 - p_{HR}^j}, \end{aligned}$$

c.f. Eq. (27) for $\bar{\beta }.$ Thus, for the parameters estimated from the data, the bifurcation cannot occur for these pairs, no matter what the value of $\beta _2$ is. In such a case the disease-free steady state is always unstable and there always exists the endemic steady state.

In Fig. 8b, we report the average yearly number of admissions to each of 163 hospitals and classify the pairs according to $\psi _i^j$ values. Clearly, condition $\psi _2 \ge \psi _1$ holds for all hospital-community pairs.

### Prevalence of multiresistant pathogens

Using the open-source SciPy library^37^, we perform numerical simulations of system (1) with parameters estimated for each of the separate 163 hospital-community pairs as presented above. Assuming that at the initial time ($t=0$) there are $8.6\%$ colonised patients in communities (c.f.^36^) and $17.2\%$, i.e. twice as many colonised patients in hospitals, for each simulation we set the following initial condition:

41 $$\begin{aligned}{} & {} \Big (S_i^j\left( 0 \right) , I_i^j\left( 0 \right) , V_i^j\left( 0 \right) , W_i^j\left( 0 \right) \Big ) =\nonumber \\{} & {} \quad (0.828 H_i^{*,j}, 0.172 H_i^{*,j}, 0.914 C_i^{*,j}, 0.086 C_i^{*,j}),\nonumber \\{} & {} \quad i=1,2, \, j=1,...,163, \end{aligned}$$

where $H_i^{*,j}, C_i^{*,j}$ are calculated according to (40).

**Figure 9:**
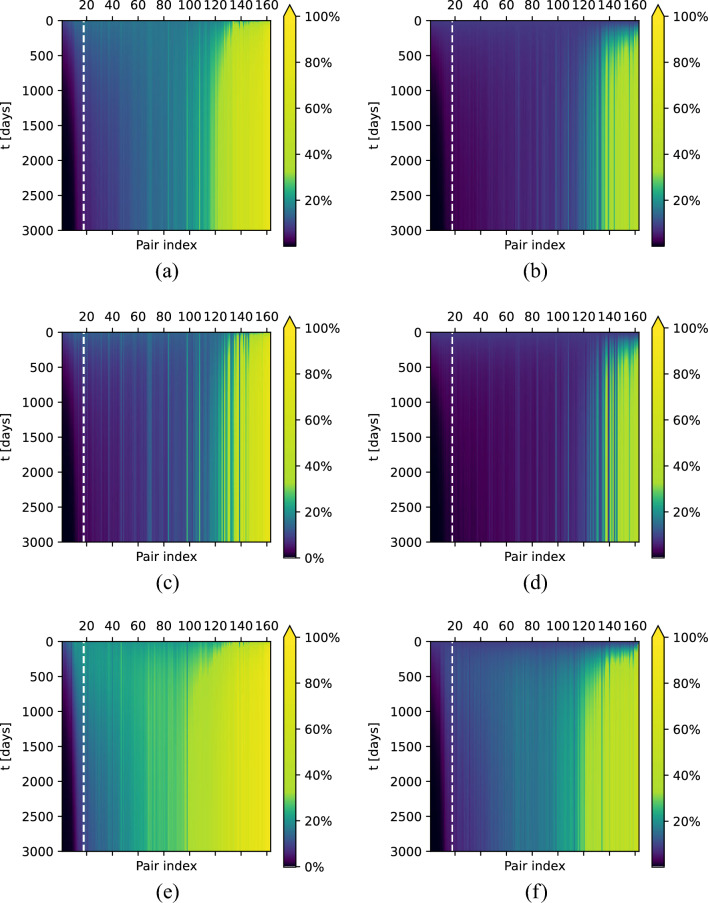
Bacteria prevalence in the hospitals (**left** column) and communities (**right** column), for (**a**,**b**) both risk groups (expressed as a percentage of $I_1^j + I_2^j$ among the whole hospital population and percentage of $W_1^j + W_2^j$ among the whole community population, respectively); (**c**,**d**) low-risk group (expressed as the percentage of $I_1^j$ among the hospital population from low-risk group and percentage of $W_1^j$ among the community population from high-risk group); (**e**,**f**) high-risk group (expressed as the percentage of $I_2^j$ among the hospital population from high-risk group and percentage of $W_2^j$ among the community population from high-risk group), over $3\,000$ days for each of the separate hospital-community pairs ($j = 1, \dots , 163$), calculated from the solutions to system (1) with initial condition (41). Hospital-community pairs are sorted in ascending order based on $\mathscr {R}_0^j$ value. Vertical dashed line indicates the first pair for which $\mathscr {R}_0^j > 1.$.

Figure 9a–f shows how the percentage of colonised patients changes over $3\,000$ days for each of the separate hospital-community pairs. In Fig. 9a,b, we illustrate the changes in bacteria prevalence within the whole population, while in Fig. 9c–f—for low-risk and high-risk groups, respectively. Plots in the left column represent the changes of the prevalence in the hospitals, whereas plots in the right column — changes in the communities.

**Figure 10:**
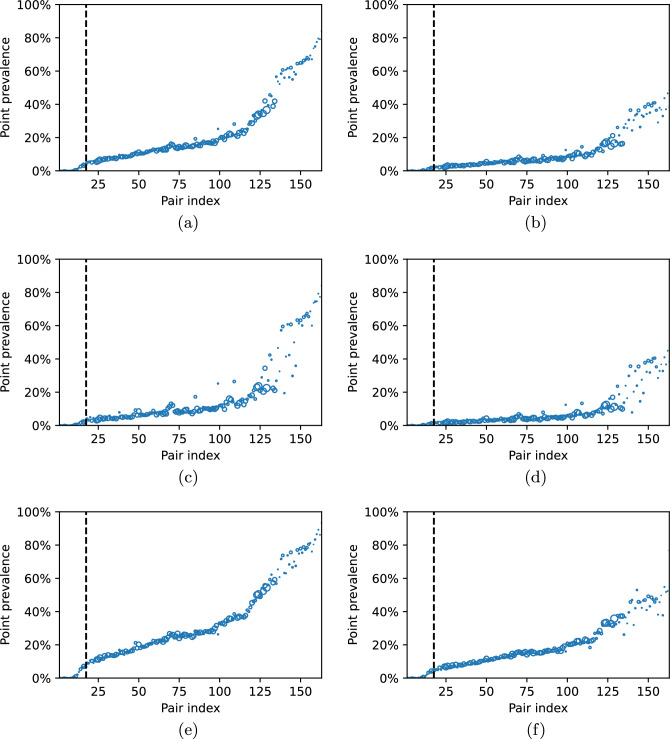
Point prevalence in the hospitals (**left** column) and communities (**right** column), for (**a**,**b**) both risk groups (expressed as a percentage of $I_1^j + I_2^j$ among the whole hospital population and percentage of $W_1^j + W_2^j$ among the whole community population, respectively); (**c**,**d**) low-risk group (expressed as the percentage of $I_1^j$ among the hospital population from low-risk group and percentage of $W_1^j$ among the community population from high-risk group); (**e**,**f**) high-risk group (expressed as the percentage of $I_2^j$ among the hospital population from high-risk group and percentage of $W_2^j$ among the community population from high-risk group), calculated at the end of the 3000 days long simulation for each of the separate hospital-community pairs ($j = 1, \dots , 163$), based on the solutions to system (1) with initial condition (41). Hospital-community pairs are sorted in ascending order based on $\mathscr {R}_0^j$ value. Vertical dashed line indicates the first pair for which $\mathscr {R}_0^j > 1.$ The size of markers is proportional to the average pair size $PS_j$.

In each figure, we observe a clear pattern that for considered hospital-community pairs with $\mathscr {R}_0 < 1$ bacteria prevalence fades over time, while for pairs with $\mathscr {R}_0 > 1$ it stabilises on some non-zero level. In addition, we observe that for the majority of hospital-community pairs with $\mathscr {R}_0 > 1,$ the prevalence in the high-risk group is much higher than in the low-risk group, see also Fig. 10a–f, where the point prevalence (at day 3000) in hospitals and communities for the cases presented in Fig. 9a–f is reported.

**Figure 11:**
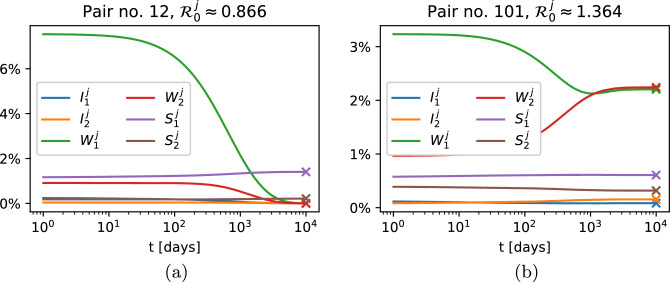
Solutions of system (18) with initial condition (41) for two selected hospital-community pairs, in which (**a**) $\mathscr {R}_0^j \approx 0.866;$ (**b**) $\mathscr {R}_0^j \approx 1.364$. Cross-marks indicate values of variables at the (**a**) disease-free steady state; (**b**) endemic steady state calculated analytically as indicated in “Mathematical properties of the single hospital-community pair model with patient risk groups” section.

In Fig. 11, we present the solutions of system (18) over $10\,000$ days for two specific hospital-community pairs (pair number 12, where $\mathscr {R}_0^{12} \approx 0.866$, and pair number 100, where $\mathscr {R}_0^{101} \approx 1.364$). Depending on whether $\mathscr {R}_0$ is greater or less than 1, we observe different dynamics of the solutions to system (18). For $\mathscr {R}_0 < 1,$ solutions converge to the disease-free steady state, while for $\mathscr {R}_0 > 1,$ they converge to the endemic steady state. This agrees with the analytical results presented in Section “Mathematical properties of the single hospital-community pair model with patient risk groups”.

In Tables 1 and 2, we present the sample Pearson correlation coefficients weighted by pair size for hospital (or community) prevalence and different characteristics of hospital-community pairs calculated using the following formula:

$$\begin{aligned} \text {corr}_w(x, y, w) = \frac{\text {cov}_w(x, y, w)}{\sqrt{\text {cov}_w(x, x, w)} \sqrt{\text {cov}_w(y, y, w)}}, \end{aligned}$$

where

$$\begin{aligned} \text {cov}_w(x, y, w) = \frac{\sum _i w_i \left( x_i - m_w \left( x, w \right) \right) \left( y_i - m_w \left( y, w \right) \right) }{\sum _i w_i} \end{aligned}$$

and *x* and *y* are the investigated variables represented by vectors, $m_w(x, w)$ is the weighted mean of vector *x* with weights vector *w* reporting the pair sizes.

Clearly, characteristics $\mathscr {R}_0$, $\psi _1$ and $\psi _2$ are strongly correlated with the prevalences, see Table 1, however, $\psi _1$ is less strongly correlated compared to $\mathscr {R}_0$ and $\psi _2$. In all cases, except parameters $\alpha _i$, correlations are positive. The strongest correlation is observed between the basic reproduction numbers and the percent of colonised individuals in the hospitals (hosp. prev. general) and between the basic reproduction numbers and the percent of colonised individuals in communities (comm. prev. general), as well as between the basic reproduction numbers and the prevalences limited to particular risk groups. Thus, in both risk groups $\mathscr {R}_0$ can be expected to be a better predictor of bacteria prevalence than the group’s $\psi _i$. We also note the fact that in terms of the absolute value, prevalences are more strongly correlated with $\varepsilon _i$ than with $\alpha _i$.

Pair sizes, $p_{HR}$, $G_2$, and $C_2^*$ reported in Table 2 do not correlate strongly with the prevalences. On the other hand, $H_1^*$ and $H_2^*$ are strongly positively correlated with the percentages of colonised individuals in both hospital and the community, whereas $C_1^*$ is the only one correlated negatively with prevalences.

**Table 1:** Pearson weighted correlation coefficients for the hospital (or community) prevalence and: basic reproduction number $\mathscr {R}_0$, basic reproduction number of a given risk group $\psi _i$, discharge rates $\alpha _i$ and admission rates $\varepsilon _i$.

	$\mathscr {R}_0$	$\psi _1$	$\psi _2$	$\alpha _1$	$\alpha _2$	$\varepsilon _1$	$\varepsilon _2$
Hosp. prev. general	0.9428	0.6796	0.8584	$-$0.5049	$-$0.4153	0.6089	0.5572
Hosp. prev. LR	0.9193	0.743	0.8493	$-$0.5005	$-$0.3851	0.6215	0.4669
Hosp. prev. HR	0.9063	0.6228	0.8195	$-$0.5161	$-$0.426	0.592	0.6105
Comm. prev. general	0.9407	0.7149	0.8585	$-$0.5059	$-$0.4012	0.634	0.5293
Comm. prev. LR	0.9067	0.7486	0.8365	$-$0.4926	$-$0.3734	0.6289	0.4488
Comm. prev. HR	0.8874	0.6266	0.8044	$-$0.5205	$-$0.4164	0.6135	0.6144

The weights correspond to the pair sizes. Hosp. prev. general stands for the fraction of colonised individuals in the hospital; Hosp. prev. LR–the fraction of colonised low-risk individuals in the hospital; Hosp. prev. HR – the fraction of colonised high-risk individuals in the hospital.

**Table 2:** Pearson weighted correlation coefficients for the hospital (or community) prevalence and: the pair sizes, hospital and community populations of risk groups *i* in a steady state, respectively $H_i^*$ and $C_i^*$, the average proportion of high-risk patients in a hospital $p_{HR}$ and the total population of the high-risk group $G_2$.

	Pair size	$H_1^*$	$H_2^*$	$C_1^*$	$C_2^*$	$p_{HR}$	$G_2$
Hosp. prev. general	0.1689	0.6265	0.7331	$-$0.3963	0.192	0.1814	0.2277
Hosp. prev. LR	0.0972	0.6934	0.6525	$-$0.3504	0.1354	0.0673	0.1687
Hosp. prev. HR	0.2281	0.5621	0.7589	$-$0.4347	0.244	0.2409	0.279
Comm. prev. general	0.1387	0.6651	0.7038	$-$0.3787	0.1671	0.1285	0.2021
Comm. prev. LR	0.0722	0.7019	0.6317	$-$0.3393	0.1237	0.0447	0.1563
Comm. prev. HR	0.2398	0.5655	0.7434	$-$0.4252	0.2347	0.2153	0.2693

The weights correspond to the pair sizes. Hosp. prev. general stands for the percent of colonised individuals in the hospital; Hosp. prev. LR – the percent of colonised low-risk individuals in the hospital; Hosp. prev. HR – the percent of colonised high-risk individuals in the hospital.

In conclusion, the simulations demonstrate that quantitatively different cases are present in the regional healthcare system for Lower Saxony, under the assumption that inter-hospital ties are neglected. Depending on the basic reproduction number, the disease either eventually vanishes ($\mathscr {R}_0 < 1$), or it becomes endemic ($\mathscr {R}_0 > 1$). Since $\mathscr {R}_0$ is derived from hospital admission/discharge statistics and pathogen transmission/recovery rates, it may be used to estimate the susceptibility of individual hospital-community pairs for a given pathogen.

### Interventions

In order to evaluate the efficiency of prevention strategies, let us first consider the relationship between basic reproduction number $\mathscr {R}_0$ and transmission rates for risk groups $\beta _1$, $\beta _2$. Clearly, from Eqs. (23), (10), (6) it follows that we have linear relationship between $\mathscr {R}_0$ and $\beta _{i}$ ($i=1,2$)

$$\begin{aligned} \mathscr {R}_0 = K_1 \beta _{1} + K_2 \beta _2, \end{aligned}$$

where $K_i=\frac{H_i^*}{(H_1^* + H_2^*)(\alpha _i + \gamma _i)(1 - q_i)}>0$ is independent of both $\beta _1$ and $\beta _2$ for $i=1,2$. Thus, the basic reproduction number is constant on lines $l=K_1 \beta _{1} + K_2 \beta _2$ for $l>0$.

**Figure 12:**
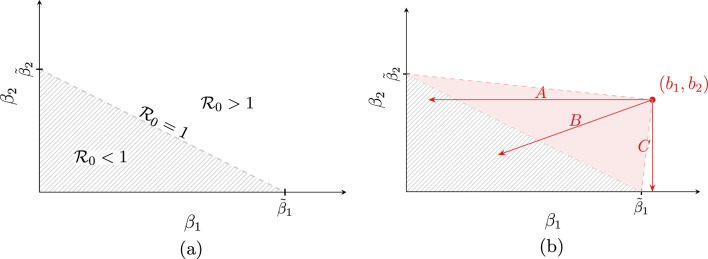
(**a**) The value of basic reproduction number as a function of risk-based transmission rates; (**b**) Examples of possible interventions *A*, *B*, *C* for initial point $(b_1,b_2)$ for which $\mathscr {R}_0>1$. The red triangle represents the area that each successful intervention must cross.

The line $\mathscr {R}_0=1$, combined with lines $\beta _1=0$ and $\beta _2=0$ are boundaries of a triangle *T* within which $\mathscr {R}_0<1$ and, consequently, disease-free steady state is globally asymptotically stable, see Fig. 12a. The hospital-level interventions are represented as the changes of one or both transmission rates. A successful eradicating intervention transforms a point from the area where $\mathscr {R}_0>1$ to the area where $\mathscr {R}_0<1$ (i.e. the triangle *T*). As presented in Fig. 12b, the intervention can impact only the first or only the second risk group (arrows *A* and *C*, respectively) or both of them at the same time (arrow *B*). In order to perform a successful eradicating intervention, the line between the initial and the final state must intersect the interior of the red triangle (Fig. 12b). In particular, it means that if $b_i\ge \tilde{\beta }_i$ then any intervention that influences only transmission rate $\beta _{3-i}$ cannot successfully eradicate the bacteria. An example of such a situation for $i=1$ is presented as point $(b_1,b_2)$ in Fig. 12b, i.e. it shows a case in which any intervention targeted to reducing the transmission among the high-risk patients only is insufficient for the complete bacteria eradication. There is an open interval of angles for which the arrow can transport the initial point to triangle *T* and it is given by $\left( \pi +\arctan \left( \frac{b_{2}-\tilde{\beta }_2}{b_{1}}\right) ,\frac{3\pi }{2}+\arctan \left( \frac{\tilde{\beta }_1-b_1}{b_{2}}\right) \right)$. So it is possible, that in some cases one of the transmission rates increases, but, nonetheless, the basic reproduction number will get less than 1. Thus, from a theoretical perspective, there can exist interventions that successfully eradicate bacteria, despite increasing transmission rate among one risk group.

**Figure 13:**
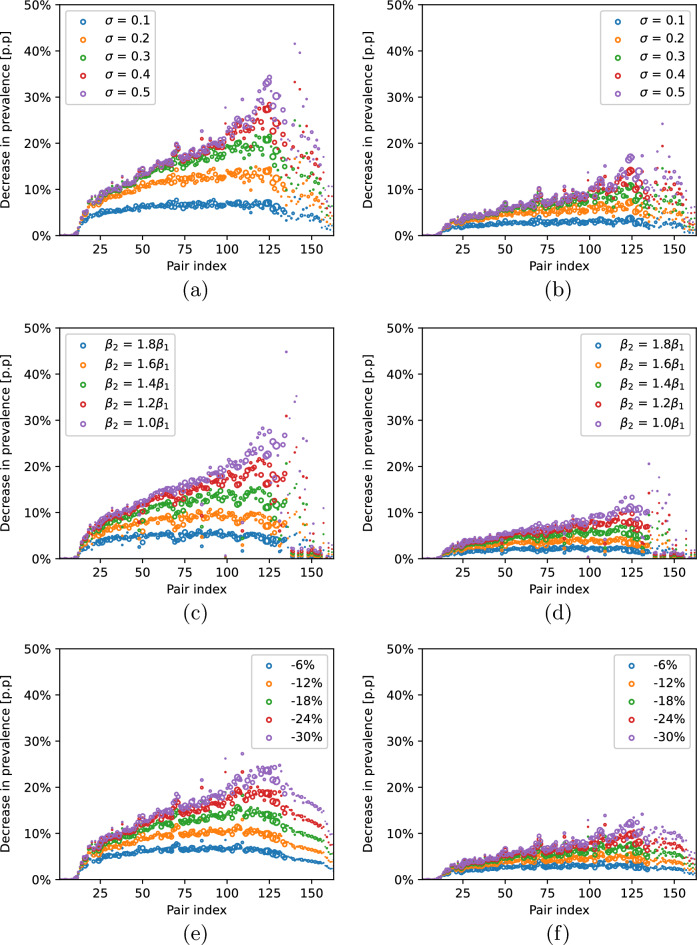
Decrease in bacteria prevalence in the hospitals (**left** column) and communities (**right** column) for both risk groups, measured in percentage points (p.p.), after intervention (**a**,**b**) introducing patient screening and thus increasing parameter $\sigma$; (**c**,**d**) reducing transmission rate in the high-risk group and thus decreasing parameter $\beta _2$; (**e**,**f**) reducing transmission rate population-wide and thus decreasing parameters $\beta _1$ and $\beta _2$ at the same rate. Prevalence is expressed as the percentage of $I_1^j + I_2^j$ among the whole hospital population and the percentage of $W_1^j + W_2^j$ among the total community node population, respectively, after 3000 days from the start of the simulation, for each of the separate hospital-community pairs ($j = 1, \dots , 163)$, calculated from the solutions to system (1) with initial condition (41). Hospital-community pairs are sorted in the same order as in 9. The size of markers is proportional to the average pair size $PS_j$.

One can also consider the introduction of more successful patient screening on admission (parameter $\sigma$ impacted). The effects of all proposed types of interventions are shown in Fig. 13, where the decrease in bacteria prevalence in the respective hospital-community pairs is presented. These three types of interventions can be scaled to have similar effectiveness. The results of different interventions in the same hospital-community pair are comparable in the pairs with pair indices less than 135. Nevertheless, the differences are more pronounced for the remaining pairs, which are characterised by the smaller average pair size $PS_j$ or the unusually high or low proportion of the high-risk patients $p_{HR}^j$, c.f. Fig. 5. Therefore, to choose the most appropriate course of the intervention, a more advanced, optimisation-based decision process is needed, which would also take the costs of the intervention into consideration.

**Figure 14:**
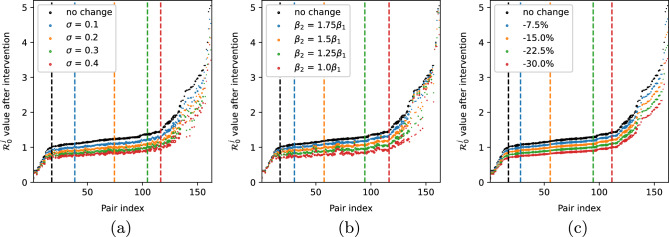
Values of $\mathscr {R}_0^j$ calculated according to formula (39) before and after applying the interventions (**a**) introducing patient screening and thus increasing parameter $\sigma$; (**b**) reducing transmission rate in the high-risk group and thus decreasing parameter $\beta _2$; (**c**) reducing transmission rate population-wide and thus decreasing parameters $\beta _1$ and $\beta _2$ at the same rate. Hospital-community pairs are sorted in the same order as in 9. Each vertical dashed line indicates the first pair for which recalculated $\mathscr {R}_0^j > 1$ – the greater the intervention, the closer the line is to the right. The size of markers is proportional to the average pair size $PS_j$.

On the other hand, Fig. 14 shows how the $\mathscr {R}_0^j$ can change in comparison with the original value when recalculated after the considered interventions. Clearly, the most extreme interventions result in the largest reduction of basic reproduction numbers. We observe the same pattern as in Fig. 5, namely two distinct groups of hospital-community pairs: one consisting of pairs indexed from 1 to around 135, where we see a similar response to the interventions, and the other with a large diversity in results. Interestingly, Fig. 14b indicates that interventions focusing on the high-risk group applied to the hospitals with a low fraction of such patients may not achieve a satisfactory effect. This is not the case for the interventions focused on raising the effectiveness of screening or interventions concerning both risk groups simultaneously, where the effects are similar for all hospital-community pairs.

## Discussion and summary

Model (1) is intended for simulations of hospital-acquired infection dynamics in a hospital and a community of patients coupled with this hospital. The model extends a previously published model^17^ by stratification into low- and high-risk patients. Depending on the basic reproduction number, two scenarios are possible: either it is low enough for there to be only a disease-free steady state, or it is high enough to indicate the simultaneous existence of an endemic steady state. Moreover, the basic reproduction number for model (1) is simply a convex combination of basic reproduction numbers of^17^ for decoupled risk groups. The mathematical analysis indicates that it is not possible to attain a mixed steady state, in which the endemic state is present in only one group (Remark 3): either it is disease-free, or endemic simultaneously in both groups. The mixed steady state would be only possible if these groups would be separated from each other.

Heterogeneity in prevalence for simulation results in different healthcare facilities is observed for both this and previous^17^ model, and for both models, it is correlated with respective basic reproduction numbers. In addition, as expected, the simulations indicate that the prevalence in the high-risk group is generally higher than in the low-risk group. On the other hand, prevalence results for communities coupled with those facilities are not representative of the communities as a whole, since during simulations only a small community subset, present in the hospital records, was considered.

Thus, the theoretical analysis, as well as simulation results, indicate that the division into low/high-risk groups does not lead to qualitatively new dynamics at the population level, whereas quantitative behaviour depends on the exact parameter values. However, new light is shed on how the pathogen transfer dynamics affects the risk groups.

The strength of model (1) lies in the capability to simulate interventions addressed to a particular risk group. For the model without risk groups, targeted interventions lead to a substantial decrease in the prevalence^19^. However, the problem to overcome is the cost of such interventions. Simulations with model (1) demonstrate how division into risk groups may lower these costs. As demonstrated in Fig. 13a,b, a great reduction of the prevalence could be achieved by screening and decolonising patients on admission. But this is hardly a viable option, as such decolonisation or perfect isolation of positively-tested patients is not possible in practice, not to mention the additional burden due to intensive initial testing. However, it is a reference point. By introducing increased preventive countermeasures aimed at high-risk patients alone it is possible to obtain similar effects as through the process of screening (c.f. Fig. 13c,d). Moreover, the high-risk group is smaller than the low-risk group in most healthcare facilities, so the interventions would be applied to only a fraction of the patients. It must be noted that despite the satisfactory reduction in the prevalence, these interventions do not guarantee a switch from the endemic state regime to the disease-free regime, as this may require a substantial reduction in transmission parameters, possibly in both risk groups (see Section “Prevalence of multiresistant pathogens”).

Some limitations of this model come from the nature of the SIS-type ODE systems, as it is assumed that all the considered populations are homogeneous within themselves and well-mixed. Additionally, when it comes to the bacteria transmission process, we assume homogeneous mixing between the risk groups in hospitals.

As mentioned before, the simulation results might not realistically depict the transmission dynamics within the entire community outside of hospitals, as the considered dataset only accounts for individuals who visited a hospital at least once during the eight-year period. Furthermore, the results presented in this study are based on hospitals decoupled from each other. In further work, it would be beneficial to extend the model to introduce direct and indirect patient transfers between hospitals and to simulate targeted interventions within one or both risk groups. In particular, it is important to determine if the interventions based on the risk groups would be more effective than the interventions ignoring them.

### Ethical approval and informed consent

The study was conducted in accordance with the Declaration of Helsinki. The analyses were performed using a pre-existing claims dataset created as part of the routine administrative activities of a statutory health insurance provider. Its scientific use is regulated by German law in the German Social Code “Sozialgesetzbuch” and the data is anonymized. The data protection officer of the Local Statutory Health Insurance of Lower Saxony-AOK Niedersachsen (Germany) has given permission for this study to use the data for scientific purposes. The project was reviewed by the ethical commission of the Medical Faculty of the Martin Luther University Halle-Wittenberg on March, 22nd 2017, which provided a written votum on March, 28th 2017 that informed consent of the patients is not required.

### Supplementary Information

Supplementary Figures.

## Data Availability

The anonymised insurance data are owned by a third party (AOK Lower Saxony) and authors do not have permission to share them. These data may be requested from AOK Lower Saxony: AOK Niedersachsen: Hildesheimer Straße 273, 30519 Hannover; https://niedersachsen.aok.de/ Numerical data for figures are given in a spreadsheet within Supplementary Material S1.
